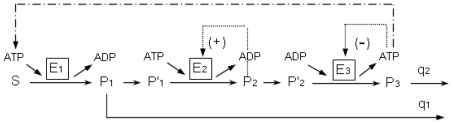# Quantitative Analysis of Cellular Metabolic Dissipative, Self-Organized Structures

**DOI:** 10.3390/ijms11093540

**Published:** 2010-09-27

**Authors:** Ildefonso Martínez de la Fuente

**Affiliations:** Institute of Parasitology and Biomedicine “López-Neyra” (CSIC), Parque Tecnológico de Ciencias de la Salud, Avenida del Conocimiento s/n, 18100 Armilla (Granada), Spain; E-Mail: mtpmadei@ehu.es; Tel.: +34-958-18-16-21; Fax: +34-958-18-16-32

**Keywords:** metabolic self-organization, dissipative structures, metabolic dynamics, systems biology, quantitative biology

## Abstract

One of the most important goals of the postgenomic era is understanding the metabolic dynamic processes and the functional structures generated by them. Extensive studies during the last three decades have shown that the dissipative self-organization of the functional enzymatic associations, the catalytic reactions produced during the metabolite channeling, the microcompartmentalization of these metabolic processes and the emergence of dissipative networks are the fundamental elements of the dynamical organization of cell metabolism. Here we present an overview of how mathematical models can be used to address the properties of dissipative metabolic structures at different organizational levels, both for individual enzymatic associations and for enzymatic networks. Recent analyses performed with dissipative metabolic networks have shown that unicellular organisms display a singular global enzymatic structure common to all living cellular organisms, which seems to be an intrinsic property of the functional metabolism as a whole. Mathematical models firmly based on experiments and their corresponding computational approaches are needed to fully grasp the molecular mechanisms of metabolic dynamical processes. They are necessary to enable the quantitative and qualitative analysis of the cellular catalytic reactions and also to help comprehend the conditions under which the structural dynamical phenomena and biological rhythms arise. Understanding the molecular mechanisms responsible for the metabolic dissipative structures is crucial for unraveling the dynamics of cellular life.

## 1. Introduction to Molecular Self-Organization in the Cellular Metabolism

Living cells are essentially dynamic metabolic systems, which are highly self-organized and formed by complex membranes surrounding a dense fluid mixture where millions of different biochemical elements interact to form self-assembled aggregates, a rich variety of supra-macromolecular functional structures and a great diversity of temporal metabolic behaviors.

The enzymes are the most outstanding molecules of these surprisingly reactive systems. They are responsible for almost all the biomolecular transformations, which globally considered are called cellular metabolism. Likewise, the dynamic functional organization of the cellular metabolism acts as an intricate network of densely integrated biochemical reactions forming one of the most complex dynamical systems in nature [1,2].

From another perspective, the cells can be considered as open systems that operate far-from-thermodynamic- equilibrium and exchange energy and matter with the external environment. A part of the energy inflow is used to produce a form of energy of higher thermodynamic value, *i.e.*, lower entropy, which allows to diminish the number of chemical entities and to increase their dimension by means of biochemical interactions and molecular bonds, emerging highly ordered macro structures and complex functional dynamic behaviors [3].

These kinds of spatial and functional molecular structures constitute a new type of supramolecular organization in the far-from-equilibrium open systems that was called dissipative structures by I. Prigogine [4].

The dissipative structure constitutes the fundamental element to understand the emergence of the spatial-functional architecture in cells and provide a conceptual framework that allows us to unify the dynamic, self-organized metabolic processes that occur in all biological organisms.

### 1.1. Supramolecular Self-Organization of the Catalytic Activities

The conditions prevailing inside the cell are characterized by a surprising molecular crowding and, in this interior medium, the enzymes do not work in an isolated way but forming molecular associations (supramolecular organization), e.g., the analysis of proteome of *Saccharomyces cerevisiae* has shown that at least 83% of all proteins form complexes containing from two to 83 proteins, and its whole enzymatic structure is formed by a modular network of biochemical interactions between enzymatic complexes [5].

Intensive studies of protein-protein interactions show thousands of different interactions among enzymatic macromolecules, which self-assemble to form large supramolecular complexes. These associations occur in all kinds of cells, both prokaryotes and eukaryotes [6–10].

Likewise, experimental observations have explicitly shown that many enzymes that operate within metabolic pathways may form functional supramolecular catalytic associations. Some of the first experimentally isolated enzymatic associations were, among others, the glycolytic subsystem [11], five enzymes from the cycle of the tricarboxylic acid [12], a triple multienzymatic-associate formed by the alpha-ketoglutarate dehydrogenase complex, the isocitrate dehydrogenase and the respiratory chain [13], and the complex formed by malate-dehydrogenase, fumarase and aspartate transferase [14]. Nowadays there are enough experimental data confirming the existence of numerous enzymatic associations belonging to metabolic routes, such as lipid synthesis, glycolysis, protein synthesis, the Krebs cycle, respiratory chain, purine synthesis, fatty acid oxidation, urea cycle, DNA and RNA synthesis, amino acid metabolism, cAMP degradation, *etc.* [15–20].

Association of various enzymes in large complexes (metabolon) allows the direct transfer of their common intermediate metabolites from the active site of one enzyme to the catalytic centre of the following enzyme without prior dissociation into the bulk solvent (substrate channeling). This process of non-covalent direct transfer of metabolic intermediates allows for a decrease in the transit time of reaction substrates, originating a faster catalysis through the pathway, preventing the loss of reaction intermediates by diffusion and increasing the efficiency and control of the catalytic processes in the multienzymatic aggregate [21–25]. Substrate channeling can occur within protein matrix channels or along the electrostatic surface of the enzymes belonging to macromolecular complex [26,27].

Different studies have shown that many enzymes that operate within metabolic pathways exhibit substrate channeling, including glycolysis, the Krebs cycle, purine and pyrimidine biosynthesis, protein biosynthesis, amino acid metabolism, DNA replication, RNA synthesis, lipid metabolism, *etc.* [28–33].

### 1.2. Structural Microcompartmentalization of the Metabolic Processes

In addition, reversible interactions of enzyme aggregates with structural proteins and membranes are a common occurrence in eukaryotic cells, which can originate the emergence of metabolic microcompartments within the soluble phases of cells [34–42].

Substrate channeling and microcompartmentalization of the cytoplasm provide high catalytic efficiency and biochemical mechanisms of great physiological importance for the control of specific enzymatic pathways and for the inter-pathway regulations.

Metabolic microcompartmentalization has been notably investigated in several eukaryotic cells, fundamentally in muscle and brain cells. In this sense, it is to highlight the works of V. Saks and colleagues on the structural organization of the intracellular energy transfer networks in cardiac cells where macromolecules, myofibrils, sarcoplasmic reticulum and mitochondria are involved in multiple structural and functional interactions, which allow the organization in the intracellular medium of compartmentalized energy transfer and other related metabolic processes. This supra structural organization has been called “intracellular energetic units” (ICEU) and represents the basic organization of muscle energy metabolism [43–50].

Similarly to what has been described for cardiac cells, it also functions in brain cells, particularly in synaptosomes [51,52].

Extensive studies of spatial metabolic structures during the last three decades have shown that the formation of functional enzymatic associations (macromolecular self-organization), the metabolite channeling and the microcompartmentalization of the metabolic processes (supra-macro-molecular organization) are the principal ways of structural organization of the eukaryotic cell metabolism.

Prokaryotic cells also exhibit microcompartments, but in this case they have outer shells which are composed of thousands of protein subunits and are filled with enzymes belonging to specific metabolic pathways in the interiors [53,54].

Contrary to eukaryotic cells, prokaryotic microcompartments do not contain lipid structures and consist of widespread compartments (about 100–200 nanometers) made of protein shells (the major constituents are proteins of the so-called “bacterial micro-compartment”) which surround and enclose different enzymes [55–59].

Although bacterial microcompartments were first observed more than 40 years ago, a detailed understanding of how they function is only now beginning to emerge [54].

The organization of cooperating enzymes into macromolecular complexes and their integration in microcompartments is a central feature of cellular metabolism, crucial for the regulation and efficiency of cellular processes and fundamental for the functional basis of cell life.

### 1.3. Metabolic Temporal Self-Organizations

The cellular organization at the molecular level presents another relevant characteristic: the emergence of functional structures which allow the temporal self-organization of metabolic processes.

A large number of experimental observations have shown that the enzymes apart from forming functional catalytic associations can exhibit oscillatory catalytic patterns (temporal self-organization).

In the far-from-equilibrium conditions prevailing inside the cell, the catalytic dynamics of enzymatic sets present transitions between different stationary and oscillatory molecular patterns. Each dissipatively structured functional enzymatic association (metabolic subsystem) acts as a catalytic entity, in which the activity is autonomous with respect to the other enzymatic associations and spontaneously organized molecular oscillations may emerge comprising an infinite number of distinct oscillatory activity regimes. When the oscillations in an enzymatic association are periodic [3,60–63], all the metabolic intermediaries oscillate with the same frequency but different amplitudes [60].

Numerous experimental observations of temporal metabolic structures both in prokaryotic and eukaryotic cells have shown the spontaneous emergence of molecular oscillations in most of the fundamental metabolic processes. For instance, there are oscillatory biochemical processes involved in: intracellular free amino acid pools [64], biosynthesis of phospholipids [65], cytokinins [66], cyclins [67], transcription of cyclins [68], gene expression [69–72], microtubule polymerization [73], membrane receptor activities [74], membrane potential [75], intracellular pH [76], cyclic AMP concentration [77], ATP [78], respiratory metabolism [79], NAD(P)H concentration [80], glycolysis [81], intracellular calcium concentration [82], the metabolism of carbohydrates [83], beta-oxidation of fatty acids [84], the metabolism of mRNA [85], tRNA [86], proteolysis [87], urea cycle [88], the Krebs cycle [89], mitochondrial metabolic processes [90], nuclear translocation of the transcription factor [91], amino acid transports [92], peroxidase-oxidase reactions [93], photosynthetic reactions [94], and protein kinase activities [95].

Oscillations represent one of the most striking manifestations of dynamic behavior, of not only qualitative but also quantitative importance, in cell metabolic systems; e.g., considering only the transcription processes, it has been reported that at least 60% of all gene expression in *S. cerevisiae* oscillate with an approximate period of 300 min [96].

These functional structures that provide the temporal self-organization of metabolism correspond to dissipative systems, and the catalytic oscillatory behaviors find their roots in the many regulatory processes that control the dynamics of the enzymes that belong to them [3,97].

The temporal organization in the metabolic processes in terms of rhythmic phenomena covers a wide time window with period lengths ranging from milliseconds [98], to seconds [99], minutes [100,101] and hours [102].

The transition from simple periodic behavior to complex oscillatory phenomena, including bursting (oscillations with one large spike and series of secondary oscillations) [103] and chaos (irregular oscillations), is often observed in metabolic behaviors [104].

Many cytological processes such as biosynthetic pathways, assembly of macrostructures, membranes and organelles, migration and cell division, require temporal organization with many simultaneous time scales [105–108], which implies that the metabolic rhythms also require an internal coordination between different enzymatic subsystems in order to maintain the spatial and temporal organization of the dynamic metabolic processes [109–112] as well as the necessity to synchronize actively these metabolic oscillations [113].

Evidence that the cells exhibit multi-oscillatory processes with fractal properties has been reported and these dynamic behaviors seem to be consistent with scale-free dynamics spanning a wide range of frequencies of at least three orders of magnitude [90].

Some temporal functional metabolic processes are not compatible with one another. In this sense, there is also evidence of the necessity for temporal compartmentalization in cells [114–117].

Furthermore, different studies have shown that many metabolic subsystems and genes oscillate as a function of the metabolic cycle, which has added another level of complexity to these kinds of functional metabolic structures [118–123].

### 1.4. Metabolic Temporal Self-Organizations with a Period of 24 Hours

Second types of temporal-functional metabolic structures are those implied in the circadian rhythms which occur with a period close to 24 hours (the exogenous period of the rotation of the earth).

Cells adapt their metabolism to the appropriate time of day synchronizing the timing of metabolic reactions with cyclic changes in the external environment [124–126].

Circadian rhythms govern a wide variety of metabolic and physiological processes in all organisms from prokaryotes to human cells [126,127].

An intimate interplay exists between circadian clocks and metabolic functions and at least 10% of all cellular transcripts oscillate in a circadian manner [128].

The molecular processes underlying circadian rhythms have been extensively studied over the past ten years and they are based on clock proteins organized in regulatory feedback loops [129,130]. More concretely, the metabolic core that regulates circadian rhythms is based on interconnected transcriptional positive and negative feedback loops in which specific clock-factors repress the transcription of their own genes [131].

In addition to transcriptional-translational feedback loops, further levels of regulation operate to maintain circadian rhythms. These include modulation of many transcriptional factors [132,133], post-transcriptional regulation [134,135], participation of kinases [136,137] and phosphatases in the modification of the clock proteins [138], post-translational modifications [139], dynamic changes in chromatin transitions [140,141], and stability of clock proteins [142].

Quantitative molecular models for circadian rhythms have been proposed to investigate their dynamic properties based on interconnected transcriptional-translational feedback loops in which specific clock-factors repress the transcription of their own genes [143].

Theoretical and experimental advances during the past decade have clarified the main molecular processes of these circadian rhythms which can be considered as a subset of metabolic rhythms with a period, defined as the time to complete one cycle of 24 hours. Likewise, there is experimental evidence that the circadian clock shares common features with the cell cycle [144] and with other cellular processes as apoptosis [145].

### 1.5. Metabolic Temporal-Spatial Self-Organizations

When spatial inhomogeneities develop instabilities in the intracellular medium, it may lead to the emergence of spatio-temporal dissipative structures which can take the form of propagating concentration waves. This dynamic behavior is closely related to temporal metabolic oscillations.

Biochemical waves are a rather general feature of cells in which are involved pH, membrane potential, flavoproteins, calcium, NAD(P)H, *etc.* They are linked to central metabolic processes and specific physiological functions, mainly with the signal transduction and intercellular communication [146].

There are several types of waves and they vary in their chemical composition, velocity, shape, intensity, and location [147–149]. Some examples are as follows: intercellular Na^+^ waves in parallel with Ca^2+^ waves [150], complex spatiotemporal patterns of redox [151], dynamic spatial organization of ATP [152,153], travelling waves of pH [154], metabolic waves of NAD(P)H [155], phase-coupled of NAD(P)H waves and calcium oscillations [156], propagation of self-organized reaction-diffusion waves of actin filament assembly during cell locomotion [157], intracellular waves of phosphatidylinositol (3,4,5)-trisphosphate (PIP_3_) [158].

In spite of its physiological importance, many aspects of the spatial-temporal dissipative structures (such as their molecular regulatory mechanisms, the relationship to the cell cycle and the temporal metabolic behaviors) are still poorly understood.

### 1.6. Global Self-Organized Metabolic Structures

The cellular organization at the molecular level presents another relevant characteristic: the emergence of global functional structures.

In 1999, the first model of a metabolic dissipative network was developed, which was characterized by sets of catalytic elements (each of them represents a dissipatively structured enzymatic association) connected by substrate fluxes and regulatory signals (allosteric and covalent modulations). These enzymatic sets of enzymes (metabolic subsystems) may present oscillatory and stationary activity patterns [159].

By means of numerical studies, a singular global metabolic structure was found to be able to self-organize spontaneously, characterized by a set of different enzymatic associations always locked into active states (metabolic cores) while the rest of metabolic subsystems presented dynamics of *on-off* changing states (structural plasticity). In this numerical first work with dissipative metabolic networks it was also suggested that the global metabolic structure could be present in all living cells.

Later studies carried out in 2004 and 2005, implementing a flux balance analysis applied to metabolic networks, produced additional evidence of the global functional structure in which a set of metabolic reactions belonging to different anabolic pathways remains active under all investigated growth conditions, forming a metabolic core, whereas the rest of the reactions belonging to different pathways are only conditionally active [160,161]. The existence of the global metabolic structure was verified for *Escherichia coli*, *Helicobacter pylori*, and *S. cerevisiae* [161,162].

The metabolic core exhibits a set of catalytic reactions always active under all environmental conditions, while the rest of the reactions of the cellular metabolism are only conditionally active, being turned on in specific metabolic conditions. The core reactions conform a single cluster of permanently connected metabolic processes where the activity is highly synchronized, representing the main integrators of metabolic activity. Two types of reactions are present in the metabolic core: the first type is essential for biomass formation both for optimal and suboptimal growth, while the second type of reactions is required only to assure optimal metabolic performance. It was also suggested that this self-organized enzymatic configuration appears to be an intrinsic characteristic of metabolism, common to all living cellular organisms [161,162].

More recently, it has been observed in extensive dissipative metabolic network simulations that the fundamental factor for the spontaneous emergence of this global self-organized enzymatic structure is the number of enzymatic dissipative associations (metabolic subsystems) [163,164].

Metabolic dissipative networks exhibit a complex dynamic super-structure which integrates different dynamic systems (each of them corresponds to different enzymatic associations dissipatively structured) and it forms a global and unique, absolutely well defined, deterministic, dynamical system, in which self-organization, self-regulation and persistent properties may emerge [165].

### 1.7. Quantitative Analysis of Functional Metabolic Structures

Theoretical and experimental data convincingly show that cellular metabolism cannot be understood if cell interior medium is considered as a homogenous solution with dispersed isolated enzymes and without any diffusion restrictions. On the contrary, cellular organisms display a rich variety of dynamics structures, both spatial and temporal, where enzymes together with other bio-molecules form complex supramolecular associations.

Each set of cooperating enzymes, dissipatively structured and integrated into macromolecular complexes and microcompartments, acts as a metabolic dynamic subsystem and they seem constitute the basic units of the cellular metabolism.

As shown below, metabolic subsystems are advantageous thermodynamically biochemical structures, which acting as individual catalytic entities forming unique, well-defined dynamical systems and their activity are autonomous with respect to the other enzymatic associations. The understanding of the elemental principles and quantitative laws that govern the basic metabolic structure of cells is a key challenge of the post-genomic era.

In this task, it becomes totally necessary to use mathematical and physical tools based on experiments. Mathematical models and non-linear dynamics tools are useful to fully grasp the molecular mechanisms of metabolic dynamical processes. They are necessary to enable the quantitative and qualitative analysis of the functional metabolic structures and also to help to comprehend the conditions under which the structural dynamical phenomena and biological rhythms arise.

Here we present an overview, within the area of Systems Biology, of how mathematical models and non-linear dynamics tools can be used to address the properties of functional dissipative metabolic structures at different organizational levels, both for simple sets of enzymatic associations and for large enzymatic networks.

Models and computational simulations, firmly based on experiments, are particularly valuable for exploring the dynamic phenomena associated with protein-protein interactions, substrate channeling and molecular microcompartmentalization processes. These procedures and methods allow to explain how higher level properties of complex molecular systems arise from the interactions among their elemental parts.

Clarifications of the functional mechanisms underlying dynamic metabolic structures as well as the study of regulation in cellular rhythms are some of the most important applications of Systems Biology. In fact, one of the major challenges in contemporary biology is the development of quantitative models for studying regulatory mechanisms in complex biomolecular systems.

Mathematical studies of metabolic processes allow rapid qualitative and quantitative determination of the dynamic molecular interactions belonging to the functional structures, and thereby can help to identify key parameters that have the most profound effect on the regulation of their dynamics.

Likewise, the advent in the field of the molecular biology of non-linear dynamics tools, such as power spectra, reconstructed attractors, long-term correlations, maximum Lyapunov exponent and Approximate Entropy, should facilitate the collection of more quantitative data on the dynamics of cellular processes.

Systems biology is fundamental to study the functional structures of metabolism, to understand the molecular mechanisms responsible for the most basic dissipative metabolic processes and will be crucial to elucidate the functional architecture of the cell and the dynamics of cellular life.

## 2. Dissipative Structures: Thermodynamic Aspects of Self-Organization

The spontaneous self-organization of metabolic processes (such as the formation of macromolecular structures and the emergence of functional patterns) is one of most relevant questions for contemporary biology.

The theoretical basis of dissipative self-organization processes was formulated by Ilya Prigogine [4]. Within the framework of this theory, a dissipative structure is an open system that operates far from thermodynamic equilibrium and exchanges energy and matter with the external environment, and as a consequence of the interchange processes, spontaneous self-organization can emerge in the system producing higher ordered spatial macro-structures and temporal-functional metabolic patterns [3,166,167].

According to these studies, the entropy of an isolated system tends to increase toward a maximum at thermodynamic equilibrium but in an open system the entropy can either be maintained at the same level or decreased (negative variation of entropy) and the overall system does not violate the Second Law. Negative variation of entropy can be maintained by a continuous exchange of materials and energy with the environment avoiding a transition into thermodynamic equilibrium.

Entropy is a quantification of randomness, uncertainty, and disorganization. Negative variation of entropy corresponds to relative order, certainty, and organization in the system. The opposite tendency for an open system which eats up energy of low entropy and dissipates energy of higher entropy to its environment may allow for the self-organization of the system.

A system capable of continuously importing free energy from the environment and, at the same time, exporting entropy (the total entropy of the system decreasing over time) was called dissipative structure. These advantageous thermodynamic systems use a part of the energy inflow to produce a new form of energy characterized by lower entropy which self-organizes the systems [168–171]. Therefore, the dissipative structure acts as a kind of energy-transforming system that uses a part of the energy inflow to produce a new form of energy which is of higher thermodynamic value (*i.e.*, lower entropy) and the negative variation of entropy corresponds to a positive variation of information [172–174] which allows increasing the complexity of the molecular organization, producing higher ordered macro structures and functional dynamic behaviors.

When a biochemical dissipative structure diminishes the number of bimolecular entities and increases their size by means of metabolic interactions and molecular bonds complex spatial macro-structures emerge in the biochemical system from simpler structures.

In the functional plane, the ordered interacting catalytic processes of the biochemical subsystem may exhibit long range correlations originating diversity of functional dynamical patterns which corresponds to ordered temporal-functional behaviors (metabolic rhythms) [3]. The mutual assistance between self-assembly and dissipative structure formation allows the self-organization of any metabolic subsystem increasing the molecular order, functionality and complexity.

A metabolic subsystem is just a dissipatively self-organized structure where a set of functionally associated enzymes adopts a new supramolecular configuration in which ordered metabolic dynamical patterns (metabolic rhythms) may arise.

As a consequence of dissipative processes, a metabolic subsystem increases its complexity generating new spatial and functional structures that did not exist before. Self-organization is also a spontaneous process, *i.e.*, the metabolic subsystem abandoned to itself is ordered in an immediate way, emerging without the necessity of an external source of information.

The problem of the emergence of self-organized structures has been studied extensively over the past sixty years and in the dissipative structures theory other important elements must be considered such as the amplification of fluctuations, non-linear interactions, bifurcations, phase transitions, complexity theory, *etc.* [175–178].

The concept of self-organization is central to the description of molecular-functional architecture of cellular live [179–189]. Dissipative molecular self-assembling and the formation of dissipative dynamic patterns are the basic fundamental elements of the bio-molecular order, functionality and complexity emergent in all living cells.

## 3. Dissipative Self-Organization and Temporal Metabolic Patterns

In the history of research on temporarily self-organized metabolic processes, the glycolytic pathway has played an important role. Its oscillatory behavior was observed, for the first time, in the fluorescent studies of yeast cells [190] and was subsequently developed in studies on cell-free extracts [191]. The confirmation of these periodic rhythms in glycolytic mediators allowed the construction of the first two simple models of those oscillatory reactive processes [192,193]. However, a qualitatively significant step was taken with the construction, in the 70’s, of the first dynamic model where the allosteric kinetics of an enzyme was explicitly considered, reflecting the important nexus existing between the molecular basis of enzymatic regulatory processes and the glycolytic oscillations [194–196]. More concretely, the main instability-generating mechanism in the yeast glycolysis is based on the self-catalytic regulation of the enzyme phosphofructokinase, specifically, the positive feed-back exerted by the reaction products, the ADP and fructose-1,6-bisphosphate [60,194,197].

As an extension of those previous studies for glycolytic oscillations based on a single positive feedback, Goldbeter and Decroly analyzed numerically the effect of two feedback loops coupled in series on a biochemical system [198]. This model represents a simple metabolic subsystem with two irreversible enzymes arranged in series and can serve as an introductory example in quantitative numerical analysis of dissipative temporal self-organization.

The diagram <1> shows how the metabolite S brought into the system at constant speed; its transformation is catalyzed by the first allosteric enzyme E_1_, which is activated by its product P_1_; the second allosteric enzyme E_2_ is also activated by its product P_2_. The removal of the product P_2_ is supposed to be linear, with a lost constant of ks.

The processes represented in the diagram are converted to differential equations describing their rates as follows:

1a. (Rate of change of [S]) = (Rate of input of [S]) − (Rate of degradation of [S]).

The explicit equation for 1a is $\frac{dS}{dt}=v-V_{\text{max}1}\Phi$.

2a. (Rate of change of [P_1_]) = (Rate of synthesis of [P_1_]) − (Rate of degradation of [P_1_]) and the corresponding equation is

$$\frac{{dP}_{1}}{dt}=V_{\text{max}1}\Phi -V_{\text{max}2}\eta$$

3a. (Rate of change of [P_2_]) = (Rate of synthesis of [P_2_]) − (Rate of removal of [P_2_]); the equation is

$$\frac{{dP}_{2}}{dt}=V_{\text{max}2}\eta -k_{s}\gamma$$

Here, V is the speed of the substrate S brought into the metabolic subsystem; V_max1_ and V_max2_ denote the maximum activities of enzymes E_1_ and E_2_; ks is the first-order rate constant for removal of P_2_; φ and η are the enzymatic rate laws for E_1_ and E_2_ developed in the framework of the concerted transition theory [199], which are described by the following functions:

$$\begin{array}{l} \Phi =\frac{\alpha (1+\alpha )(1+\beta^{2})}{\left[{\text{L}}_{1}{\left(1+\alpha \right)}^{2}+{\left(1+\beta \right)}^{2}\right]}\\ \eta =\frac{\beta (1+\text{d}\beta ){\left(1+\gamma \right)}^{2}}{\left[{\text{L}}_{2}+{\left(1+\text{d}\beta \right)}^{2}+{\left(1+\gamma \right)}^{2}\right]} \end{array}$$

where, α, β and γ denote the normalized concentrations of S_1_, P_1_ and P_2_, divided respectively, by the Michaelis constants of E_1_ (Km1) and by the dissociation constant of P_1_ for E_1_ (K P_1_) and of P_2_ for E_2_ (KP2); L_1_ and L_2_ are the allosteric constants of E_1_ and E_2_.

Once the different elements of the equations are normalized, the time-evolution of the metabolic subsystem is described in any instant by the following three differential equations:

(1) $$\{\begin{array}{l} \frac{d\alpha }{dt}=(\text{v}/{\text{K}}_{\text{m}1})-\sigma_{1}\Phi \\ \frac{d\beta }{dt}=q_{1}\sigma_{1}\Phi -\sigma_{2}\eta \\ \frac{d\gamma }{dt}=q_{2}\sigma_{2}\eta -k_{s}\gamma \end{array}$$

σ_1_ and σ_2_ correspond to the normalized maximum activity of the enzymes E_1_, E_2_, (they are divided by the constants K_m1_, K_m2_, the Michaelis constants of the enzymes E_1_ and E_2_, respectively); q_1_ = K_m1_/K_p1_, q_2_ = K_p1_/K_p2_ and d = K_p1_/K_m2_.

Once the values for the parameters are specified and given initial values for the dependent variables (see [198] for more details), the equation can be solved numerically on a computer by means of any integration program of ordinary differential equations (ODE). In the analysis, the ks value (the removal kinetic constant for the product P_2_) was fixed as the control parameter of the multienzymatic instability-generating reactive system.

**Figure 1:**
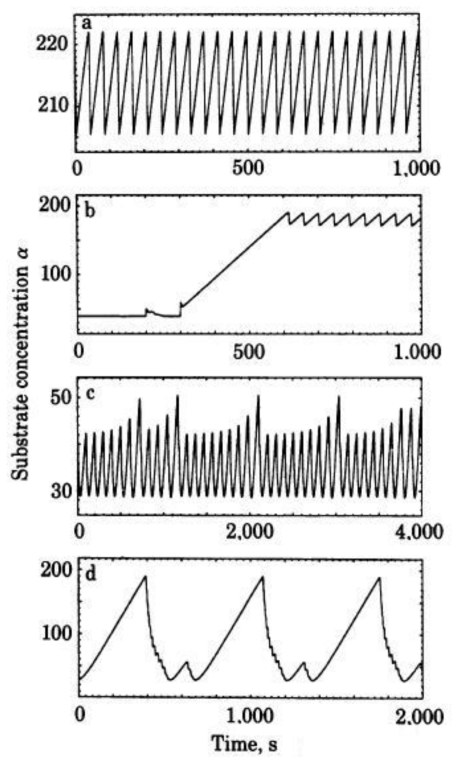
Diversity dynamic behaviors emerge in the simple dissipative metabolic subsystem. (**a**) Periodic pattern. (**b**) Hard excitation, the integral solutions depending on the initial conditions settle on two regimens: a stable steady state and a stable periodic oscillation. (**c**) Chaotic oscillations. (**d**) Complex periodic behaviors. The substrate concentration α is represented as a function of the time in seconds. Reproduced with permission from PNAS [198].

After the numerical integration, a wide range of different types of dynamic patterns can be evidenced as a function of the control parameter value. At very small ks values, the system (1) admits a single steady-state solution and when ks is increased, the steady state become unstable, leading to the emergence of a periodic pattern (Figure 1a). In this case, since the enzymatic sets exhibit a rhythmic behavior, all the metabolic intermediaries (S_1_, P_1_ and P_2_) oscillate with the same frequency but different amplitudes.

In the interval 0.792 < k_S_ ≤ 2.034 0. the metabolic subsystem exhibits the most interesting dynamical behaviors.

For instance, one can observe how for 0.792 < k_S_ ≤ 1.584 hard excitation emerges in the functional enzymatic association and two kinds of integral solutions coexist under the same control parameter value: a stable steady state and a stable periodic oscillation (bistability). The metabolic subsystem starts from a stable steady state but evolves to a stable periodic regime when the initial concentrations of S_1_, P_1_ and P_2_, exceed a determinate threshold value (Figure 1b).

At further increases in ks (1.584 < k_S_ ≤ 1.82) the metabolic subsystem undergoes a reorganization of its dynamics and spontaneously presents a temporal structure characterized by the coexistence of two stable periodic behaviors under the same control parameter value (bistability). The integral solutions now settle on two regular oscillatory regimens depending on the initial conditions (the concentrations of S_1_, P_1_ and P_2_).

Between 1.82 < k_S_ ≤ 1.974 the biochemical system exhibits one simple periodic pattern (oscillation of period-1 with one maximum and one minimum per oscillation) and when the control parameter increases (1.99 < k_S_ ≤ 2.034) the numerical solutions of the biochemical oscillator display a classical period-doubling cascade preceding chaos, *i.e.*, when ks reaches a threshold, the oscillation of period-1 becomes unstable, which leads to the establishment of a new regular oscillation of period-2 (two maximums and two minimums per oscillation); when k_S_ increases, a new instability provokes the emergence of regular oscillations of period-4; next a new bifurcation of period-8 appears, *etc.*; this cascade of bifurcations of period doubling continues successively ending in a chaotic response (this process is called Feigenbaum route).

In chaotic conditions, all the metabolic intermediaries (S_1_, P_1_ and P_2_) present infinite transitions, modifying uninterruptedly their activity so that they never repeat themselves for arbitrarily long time periods (Figure 1c).

Lastly, as ks increases beyond 2.034, complex periodic oscillations emerge in the multienzymatic subsystem (Figure 1d).

The quantitative numerical analysis of the system (1) allows showing how metabolite concentrations of the biochemical oscillator, formed by only two irreversible enzymes, vary second by second following a notable diversity of dynamic patterns as a function of the control parameter values (and the initial conditions when two dynamic behaviors coexist). In the numerical analyses, the feedback processes are the main sources of nonlinearity that favor the occurrence of instabilities which provoke the emergence of different dynamical patterns.

In the course of time, open enzymatic systems that exchange matter and energy with their environment exhibit a stable steady state. This stationary non-equilibrium state is more ordered that the equilibrium state of the same energy. Once the enzymatic subsystem operates sufficiently far-from-equilibrium due to the nonlinear nature of its kinetics, the steady state may become unstable leading to the establishment of other dynamical behavior. New instabilities may originate the emergence of different biochemical temporal behaviors.

All these dynamic patterns (including chaos) correspond to ordered motions in the system representing examples of non-equilibrium self-organizations and can therefore be considered as temporal dissipative structures.

The emergence of quantitative behaviors belonging to different metabolic subsystems has been investigated in extensive studies, mainly carried out by means of systems of differential equations, e.g., in the Krebs cycle [200], amino acid biosynthetic pathways [201], oxidative phosphorylation [202,203], glycolytic subsystem [204], transduction in G-protein enzyme cascade [205], gene expression [206], cell cycle [207], RNA silencing pathway [208], signal transduction [209], Wnt-pathway [210], fatty acid metabolism [211], DNA base excision repair [212], interferon-β induced signaling pathway [213], NF-kB metabolic subsystem [214], sphingolipid metabolism [215] and oxide/cGMP pathway [216]. These studies also show how each metabolic subsystem forms a unique, absolutely well-defined, deterministic, dynamical system.

## 4. Metabolic Self-Organization and the Cell-Cycle

Cell-cycle in eukaryotic cells is governed by a complex network of metabolic reactions controlling the activities of M-phase-promoting factors. These metabolic reactions belonging to a set of enzymes functionally associates can be self-organized in far-from-equilibrium conditions, exhibiting periodic oscillations which govern the cell-cycle.

The network forms a metabolic subsystem that mainly involves enzymes of covalent regulation and protein kinases (Cdk) whose activities depend on binding to cyclins. More concretely, mitosis-promoting factor (MPF) has been identified as a dimmer of two distinct protein molecules: a cyclin subunit and a cyclin-dependent protein kinase (Cdc2), which is periodically activated and inactivated during the cell cycle. MPF activity is regulated by synthesis and degradation of cyclin subunit and by phosphorylation and desphosphorylation of the protein kinase Cdc2 at an activatory threonine (Thr) residue and an inhibitory tyrosine (Tyr) residue. When the active form of MPF is phosphorylated on Thr161, then M-phase begins by phosphorylating a suit of target proteins involved in the main events of mitosis [217].

**Figure 2:**
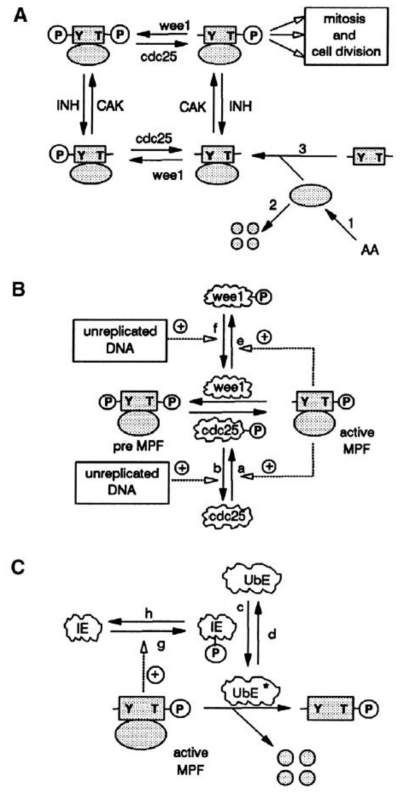
Molecular processes for M-phase control in eukaryotic cells. (**a**) Cdc2 protein kinase monomers combine with cyclin subunits to form dimers. Subunits of kinase Cdc2 can be phosphorylated and desphosphorylated at an activatory threonine (Thr) residue or/and an inhibitory tyrosine (Tyr) residue. All cyclin subunits can be degraded by an ubiquitin pathway. (**b**) Active MPF stimulates its own production, which is positive feedback. (**c**) But active MPF also stimulates the destruction of cyclin, which is negative feedback. Reproduced with permission from the Company of Biologistd Ltd. [218].

In 1991, John J. Tyson and colleagues constructed a dynamic mathematical model for cell-cycle regulation in *Xenopus* oocytes [217–219] and the predictions were subsequently confirmed in a series of recent experimental works [220–222]. The main molecular elements of the model for M-phase control can be seen in Figure 2 [218].

First, cyclin subunits synthesized from amino acids (step 1 of the Figure 2A), combine with free Cdc2 protein kinase monomers to form Cdc2-cyclin dimers (step 3). The cyclin subunits (free or bound) can be degraded by an ubiquitin pathway (step 2).

The activity of dimers can be regulated by altering the phosphorylation state by means of two kinase-phosphatase pairs: Wee1/Cdc25, which acts at Tyr15 (Y), and CAK/INH, which acts at Thr161 (T). As a consequence, the dimers can exist in four different phosphorylation states (Figure 2B).

The molecular model shows two experimentally recognized feedback loops [2]. Active MPF stimulates its own production, which is positive feedback that allows activating Cdc25 and inhibiting Wee1 (Figure 2B) but, on the other hand, active MPF stimulates the destruction of cyclin, which is negative feedback (Figure 2C).

Each molecular process represented in Figure 2 is converted to a differential equation describing its rates of synthesis and degradation as follows:

$$\left(\begin{array}{l} \text{Rate of change}\\ \text{of }[\text{total cyclin}] \end{array}\right)=\left(\begin{array}{l} \text{Rate of synthesis}\\ \text{of free cyclin} \end{array}\right)-\left(\begin{array}{l} \text{Rate of}\\ \text{deg}\;\text{radation} \end{array}\right)-\left(\begin{array}{l} Rate\;of\;\text{association}\\ with\;Cdc2\;monomers \end{array}\right)$$

The explicit equation for these processes is

$$\frac{\text{d}}{\text{dt}}[\text{total cyclin}]={\text{k}}_{1}[\text{AA}]-{\text{k}}_{2}[\text{total cyclin}]-{\text{k}}_{3}[\text{cyclin}][\text{Cdc}2]$$

A similar procedure continues until reaching a complete set of 10 equations that describe how the molecular element of the metabolic model changes with time.

1. $\frac{d[cyclin]}{dt}=k_{1}[AA]-k_{2}[cyclin]-k_{3}[cyclin][Cdc2]$
2. $\frac{\text{d}[-\text{cyclinCdc}2-]}{\text{dt}}={\text{k}}_{\text{INH}}[-\text{cyclinCdc}2-\text{P}]-({\text{k}}_{\text{wee}}+{\text{k}}_{\text{CAK}}+{\text{k}}_{2})[-\text{cyclinCdc}2-]+{\text{k}}_{25}[\text{P}-\text{cyclinCdc}2-]+{\text{k}}_{3}[\text{cyclin}][\text{Cdc}2]$
3. $\frac{\text{d}[\text{P}-\text{cyclinCdc}2-]}{\text{dt}}={\text{k}}_{\text{wee}}[-\text{cyclinCdc}2-]-({\text{k}}_{\text{25}}+{\text{k}}_{\text{CAK}}+{\text{k}}_{2})[\text{P}-\text{cyclinCdc}2-]+{\text{k}}_{\text{INH}}[\text{P}-\text{cyclinCdc}2-\text{P}]$
4. $\frac{\text{d}[\text{P}-\text{cyclinCdc}2-\text{P}]}{\text{dt}}={\text{k}}_{\text{wee}}[-\text{cyclinCdc}2-\text{P}]-({\text{k}}_{\text{wee}}+{\text{K}}_{\text{25}}+{\text{k}}_{2})\;[\text{P}-\text{cyclinCdc}2-\text{P}]{-\text{k}}_{\text{CAK}}[\text{P}-\text{cyclinCdc2}-]$
5. $\frac{\text{d}[-\text{cyclinCdc}2-\text{P}]}{\text{dt}}={\text{k}}_{\text{CAK}}[-\text{cyclinCdc}2-]-({\text{k}}_{\text{INH}}+{\text{k}}_{\text{wee}}+{\text{k}}_{2})[-\text{cyclinCdc}2-\text{P}]+{\text{k}}_{25}[\text{P}-\text{cyclinCdc}2-\text{P}]$
6. $\frac{\text{d}[\text{Cdc}2]}{\text{dt}}={\text{k}}_{\text{2}}[-\text{cyclinCdc}2-]+[\text{P}-\text{cyclinCdc}2-]+[\text{P}-\text{cyclinCdc}2-\text{P}]+[-\text{cyclinCdc}2-\text{P}]+{\text{k}}_{3}[\text{cyclin}]\;[\text{Cdc}2]$
7. $\frac{\text{d}[\text{cdc}25-\text{P}]}{\text{dt}}=\frac{{\text{k}}_{\text{a}}\left\lfloor \text{active MPF}\right\rfloor \;([\text{total cdc}25]-[\text{cdc}25-\text{P}])}{{\text{k}}_{\text{a}}+([\text{total cdc}25]-[\text{cdc}25-\text{P}])}-\frac{{\text{k}}_{\text{b}}[\text{PPase}][\text{cdc}25-\text{P}]}{{\text{k}}_{\text{b}}+[\text{cdc}25-\text{P}]}$
8. $\frac{\text{d}[\text{wee}1-\text{P}]}{\text{dt}}=\frac{{\text{k}}_{\text{e}}\left\lfloor \text{active MPF}\right\rfloor ([\text{total wee}1]-[\text{wee}1-\text{P}])}{\text{k }[\text{total wee}1]-[\text{wee}1-\text{P}]}-\frac{{\text{k}}_{\text{f}}[\text{PPase}]\;[\text{wee}1-\text{P}]}{{\text{k}}_{\text{f}}+[\text{wee}1-\text{P}]}$
9. $\frac{\text{d}[\text{IE}-\text{P}]}{\text{dt}}=\frac{{\text{k}}_{\text{g}}\left\lfloor \text{active MPF}\right\rfloor \;([\text{total IE}]-[\text{IE}-\text{P}])}{{\text{k}}_{\text{g}}+[\text{total IE}]-[\text{IE}-\text{P}]}-\frac{{\text{k}}_{\text{h}}[\text{PPase}]\;[\text{IE}-\text{P}]}{{\text{k}}_{\text{h}}[\text{IE}-\text{P}]}$
10. $\frac{\text{d}[\text{Ube}*]}{\text{dt}}=\frac{{\text{k}}_{\text{C}}[\text{IE}-\text{P}]\;([\text{total Ube}]-[\text{Ube}*])}{{\text{k}}_{\text{C}}\;+[\text{total Ube}]-[\text{Ube}*]}-\frac{{\text{k}}_{\text{d}}[\text{anti}-\text{IE}]\;[\text{Ube}*]}{{\text{k}}_{\text{d}}+[\text{Ube}*]}$

The rate constants k_25_, k_wee_ and k_2_ are defined as:

$$\begin{array}{l} {\text{k}}_{25}={\text{V}}_{25}^{\prime }([\text{total cdc}25]-[\text{cdc}25-\text{P}])+{\text{V}}_{25}*[\text{cdc}25-\text{P}]\\ {\text{k}}_{\text{wee}}={\text{V}}_{\text{wee}}^{\prime }[\text{wee1}-\text{P}]+{\text{V}}_{\text{wee}}*([\text{total wee1}]-[\text{wee}1-\text{P}])\\ {\text{k}}_{2}={\text{V}}_{2}^{\prime }([\text{total Ube}]-[\text{Ube}*])+{\text{V}}_{2}*[\text{Ube}*] \end{array}$$

The first six differential equations follow mass-action kinetics and the next four follow Michaelis-Menten kinetics.

Once the values for the 31 parameters (Michaelis constants, total enzyme levels, *etc.*) are specified and given initial values for all time-dependent variables (see [218] for more details), the equations can be solved numerically.

**Figure 3:**
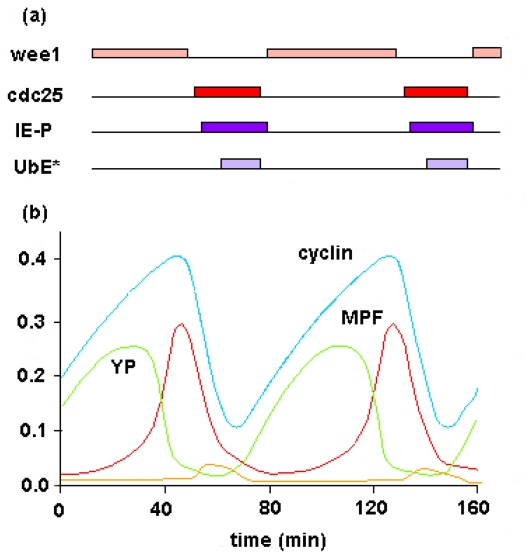
Quantitative analysis of the M-phase control system showing spontaneous periodic oscillations in the metabolic intermediaries. The total cyclin concentrations (blue), active form of MPF (red), tyrosine-phosphorylated dimers, YP, (green) and total phosphorylated cdc2 monomers (orange) are represented as a function of the time in minute. The bar graphs indicate the periods during which the active forms exceed 50% of the total amount. Reproduced with permission from the Company of Biologists Ltd. [218].

The quantitative analysis shows a main relevant behavior; stable regular oscillations emerge in the dynamic system and all the metabolic intermediaries of the metabolic subsystem oscillate with the same frequency but different amplitudes. Figure 3 shows the dynamic behaviors belonging to the concentration of total cyclin, the active form of MPF and tyrosine-phosphorylated dimers (YP) at any given instant of time. The cascade of phosphorylation and dephosphorylation involving cyclin and Cdc2 kinase is functionally self-organized in time and produces higher ordered activity patterns.

**Figure 4:**
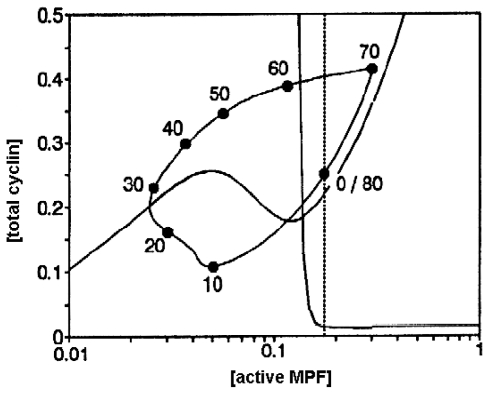
A limit cycle attractor governs the cell cycle. The cell cycle is controlled by a dynamical structure called “limit cycle” which is a closed orbit corresponding to the oscillations with a period of 80 minutes. The numbers along the limit cycle represent time in minutes after exit from mitosis. Reproduced with permission from the Company of Biologists Ltd. [218].

In Figure 4, the dynamic solutions of the system are projected onto the phase plane (the x-axis is the concentration of active MPF and the y-axis is the concentration of total cyclin) and the temporal development of the molecular network can be envisaged as the movement of the “state point” through the phase space. The closed orbit is an attractor of type limit cycle which governs the sustained oscillations in [active MPF] and [total cyclin] with a period of 80 minutes.

The cell cycle seems to be controlled by this dynamic structure (attractor) which represents the set of all the possible asymptotic behaviors and corresponds to the ordered motions in the metabolic subsystem.

The catalytic elements implicated in the cell-cycle regulation represent a group of functionally associated and dissipatively structured enzymes that form a catalytic entity as a whole. The catalytic activity of the metabolic subsystem is autonomous with respect to the other enzymatic associations which operate within far-from-equilibrium conditions and as a consequence molecular periodic oscillations spontaneously emerge. This set of dissipatively structured enzymatic associations is an absolutely well-defined, deterministic, dynamical system responsible for the control of the activities of M-phase-promoting factors.

The catalytic elements implicated in the cell-cycle regulation represent a group of functionally associated and dissipatively structured enzymes that form a catalytic entity as a whole. The catalytic activity of the metabolic subsystem is autonomous with respect to the other enzymatic associations which operate within far-from-equilibrium conditions and as a consequence molecular periodic oscillations spontaneously emerge. This set of dissipatively structured enzymatic associations is an absolutely well-defined, deterministic, dynamical system responsible for the control of the activities of M-phase-promoting factors.

For almost two decades, the initial model of Tyson has been developed with new molecular and dynamic factors as for example, bistability [223], hysteresis [224], intrinsic noise caused by molecular fluctuations [225] and the activities of hundreds of ‘executor’ proteins (EPs) [226].

## 5. Quantitative Analysis in Metabolic Networks

During the past two decades, different mathematical models have allowed for an intensive study of metabolic processes formed by large groups of enzymes including global metabolic systems.

Traditional models have focused on the kinetics of multi-enzyme systems by solving systems of differential equations and algebraic equations [227]. Petri’s net theory, among other methodologies [228], has been applied to modeling metabolic pathways [229], decomposition of large metabolic networks into smaller subnetworks [230] and topological analysis of enzymatic groups [231]. Large networks present many connections between the nodes, and their degree distributions follow a power law, so they can be considered as scale-free [232,233]. The presence of “small-world” features [234] in scale-free networks has been studied [235,236]. Constraint-based modeling approaches, such as flux-balance analysis, have been applied in several metabolic networks [160,237]. Other mathematical models have been proposed to organize the networks both in their modular and hierarchical structure [238–241].

Until recently, metabolic networks formed by enzymes and pathways have been studied individually. However, at present, mathematical models based on experimental data are aiming to integrate cellular metabolism as a whole [160–165]. Likewise, a considerable number of global genome-scale reconstructions of metabolic systems have been published in recent years [242–247].

Among the different mathematical models focused on enzymatic networks, Flux Balance Analysis (FBA) has emerged as an effective means to analzse metabolic networks in a quantitative manner demonstrating reasonable agreement with experimental data [248,249]. This method has been proved highly successful to calculate the relative flux values of metabolic reactions, as well as analyze the global cellular metabolism [160,161].

The FBA method allows finding optimal steady state flux distributions in a metabolic network subject to additional constraints on the rates of the reaction steps. FBA is based on the assumption that the dynamic mass balance of the metabolic system can be described using a stoichiometric matrix, and relating the flux rates of enzymatic reactions to the time derivatives of metabolite concentrations in the following form:

$$\frac{\text{dX}}{\text{dt}}=\text{Sv}$$

where X is an *m* dimensional column vector defining the quantity of the metabolites of a network, v is the column vector of *n* metabolic fluxes and S is the *m* × *n* stoichiometric matrix [250].

The FBA method is based on the assumption that the concentration of all cellular metabolites must satisfy the steady-state constraint and therefore the dynamic mass balance of the metabolic system must equal zero:

$$\frac{\text{dX}}{\text{dt}}=\text{Sv}=0$$

The main element of the FBM is the stoichiometric matrix, S, which describes all the biochemical transformations in a network in a self-consistent and chemically accurate matrix format. The rows of S correspond to various network components, while the columns of S delineate the reactions, or the way in which these components interact with one another [251,252], see an example hereinafter. Much progress has been made in the metabolic reconstruction process and a growing number of published stoichiometric matrices are now available [253–257].

Because most metabolic systems are underdetermined *i.e.*, there exist more unknown fluxes than equations, an objective function is used to obtain a solution by using linear programming or other optimization methods. In fact, a central challenge in FBA is to define, for a given enzymatic system, an objective function for which that system optimizes. Several linear programming strategies have been proposed to generate flux distributions that are optimized toward a particular objective function, subject to a set of governing constraints [258–260].

Typically, the maximization of the growth flux is used as the objective function [261–263], where the growth flux can be defined in terms of the biosynthetic requirements. Other examples of objective functions used in the literature include: maximizing or minimizing the rate of production of a particular metabolic product [264–267], maximizing or minimizing the rate of nutrient uptake [268], and maximizing or minimizing ATP production [269].

The development of a flux balance analysis requires the definition of all the metabolic reactions and metabolites. For example, let us consider a simple metabolic network (diagram < 2>) formed by two enzymes and comprising three metabolites (A, B and C) with two internal enzymatic processes including one reversible reaction (the fluxes R1, R2 and R3) and three exchange fluxes with one reversible reaction (R4, R5, R6 and R7) [250]:

Mass balance equations for all reactions and transport processes are written by

$$\frac{\text{dX}}{\text{dt}}=\text{Sv}$$

Reactions can be represented as an S stoichiometric matrix form with

$$\text{S}=\begin{array}{c} \text{A}\\ \text{B}\\ \text{C} \end{array}\left[\begin{array}{ccccccc} \text{R}1 & \text{R}2 & \text{R}3 & \text{R}4 & \text{R}5 & \text{R}6 & \text{R}7\\ -1 & 0 & 0 & 1 & 0 & 0 & 0\\ 1 & -1 & 1 & 0 & -1 & 0 & 0\\ 0 & 1 & -1 & 0 & 0 & -1 & 1 \end{array}\right]$$

At steady-state, Sv = 0, a set of algebraic constraints on the reactions rates can be assumed: the objective function is max Z = v_5_ (for example), and then the constraints are

$$\begin{array}{c} \text{A}\\ \text{B}\\ \text{C} \end{array}\left[\begin{array}{ccccccc} \text{R}1 & \text{R}2 & \text{R}3 & \text{R}4 & \text{R}5 & \text{R}6 & \text{R}7\\ -1 & 0 & 0 & 1 & 0 & 0 & 0\\ 1 & -1 & 1 & 0 & -1 & 0 & 0\\ 0 & 1 & -1 & 0 & 0 & -1 & 1 \end{array}\right]\left[\begin{array}{c} {\text{v}}_{1}\\ \vdots \\ {\text{v}}_{7} \end{array}\right]=0\;\;\;0\leq {\text{v}}_{1},\cdots ,{\text{v}}_{7}\leq 10$$

Once the problem of optimization is formulated, techniques of operation research can be used to obtain a solution. In this case, the optimal value of v_5_ was found to be 10.0 (see [250] for more details) with a vector of fluxes of v = [6.67 3.33 6.67 6.67 10.0 3.33 6.67]^T^.

The optimal distribution of all fluxes is:

Although classical FBA assumes steady-state conditions, several extensions have been proposed in recent years to improve the predictive ability of this method, e.g., gene regulatory constraints were incorporated into metabolic models leading to a modification of FBA called regulatory flux balance analysis (rFBA) in which Boolean rules are considered on an existing stoichiometric model of gene expression metabolism [270–272]; in the study of complex metabolic networks, an extension called energy balance analysis (EBA) incorporates the general principles of thermodynamics [273]; more recently, a regulatory matrix, called R, was developed for the representation of transcriptional regulatory networks (TRNs) (the matrix R is similar to S in that its rows and columns correspond to network components and interactions, respectively) [274]; a variant of FBA called dynamic flux balance analysis (DFBA) provides a framework for assessing the transience of metabolism due to metabolic reprogramming; this DFBA method was implemented in a dynamic optimization approach that required solving a nonlinear programming (NLP) and a static optimization approach that required using linear programming strategies [275]; finally, a recent extension of DFBA called integrated dynamic flux balance analysis (idFBA) enables the dynamic analysis of integrated biochemical networks [276].

As pointed out in the introduction section, several studies performed using metabolic networks have shown that enzymes can present a self-organized global functional structure characterized by a set of enzymes which are always in an active state (metabolic core), while the rest of the molecular catalytic reactions exhibit *on-off* changing states [160–162].

The existence of the global metabolic structure was verified for *E. coli*, *H. pylori*, and *S. cerevisiae* implementing flux balance analysis [160–162]. By means of constraint-based studies applied to metabolic networks, E. Almaas, A.L. Barabási and their group of researchers, have also shown that most current antibiotics may interfere with the metabolic core [161] and they suggest that this global organization of the cellular metabolism “probably represents a universal feature of metabolic activity in all cells, with potential implications for metabolic engineering.” This global cellular metabolic structure seems to be an intrinsic characteristic of metabolism, common to all living cellular organisms [159–165].

## 6. Effect of the Delays on Temporal Self-Organizations

Many of the metabolic dynamic analyses have ignored the impact of time delays on enzymatic oscillators, which are due to different biochemical processes such as oscillatory phase-shifts, transport, translation, translocation, and transcription.

What most of these non-linear dynamic studies in metabolic systems have in common is that have been performed through ordinary differential equations (ODE). According to this modeling, selforganized dynamic behaviors are considered to depend on the different values achieved by the parameters linked to the dependent variables. Moreover, the initial conditions are always constant values (never initial functions) and when determining the particular solutions, only a small number of freedom degrees are available, as a result of the restrictions of the ODE systems.

Within the framework of dynamical systems theory delay processes can be approximated accurately by augmenting the original variables with other auxiliary functional variables. By means of these systems of functional differential equations with delay it is possible to take into account initial functions (instead of the constant initial values of ODE systems) and to analyze the consequences that the variations in the parametric values linked to the independent variable (time) have upon the integral solutions of the system.

A typical ODE system is the following:

(2) $$\{\begin{array}{l} {\text{y}}_{1}^{\prime }(\text{t})={\text{f}}_{1}({\text{z}}_{1}(\text{t}),\ldots ,{\text{y}}_{\text{n}}(\text{t}))\\ \vdots \\ {\text{y}}_{\text{n}}^{\prime }(\text{t})={\text{f}}_{\text{n}}({\text{z}}_{1}(\text{t}),\ldots ,{\text{y}}_{\text{n}}(\text{t})) \end{array}$$

and a dynamic model governed by a delayed functional differential equations system, can take the following particular form:

(3) $$\{\begin{array}{l} {\text{y}}_{1}^{\prime }(\text{t})={\text{f}}_{1}({\text{z}}_{1}(\text{t}),\ldots ,{\text{z}}_{\text{r}}(\text{t}){\text{y}}_{\text{r}+1}(\text{t}),\ldots ,{\text{y}}_{\text{n}}(\text{t}))\\ \vdots \\ {\text{y}}_{\text{n}}^{\prime }(\text{t})={\text{f}}_{\text{n}}({\text{z}}_{1}(\text{t}),\ldots ,{\text{z}}_{\text{r}}(\text{t}){\text{y}}_{\text{r}+1}(\text{t}),\ldots ,{\text{y}}_{\text{n}}(\text{t})) \end{array}$$

where the dependent variable is a n-dimensional vector of the form y = (y_1_,...,y_n_), t being the independent variable, and the z_i_ variables appear delayed, that is z_i_(t) = h_i_(y_i_(t − λ_i_)) where λ_i_ are the corresponding delays and h_i_ are given functions. Hereafter, the z_i_ will be named functional variables.

In system (3), the derivatives of y_1_,...,y_n_, evaluated in t are related to the variables y_1_,...,y_r_ evaluated in t − λ_i_, and related to the variables y_r+1_,...,y_n_ evaluated in t.

As *y*_1_′(*t*),···, *y_n_*′ (*t*) depends of the values of y_1_,...,y_n_ in times before t, the initial conditions cannot be simply the values of y_1_,...,y_n_ in a unique time, but in an interval [t_0_−δ,t_0_] with δ = max {λ_1_,…, λ_r_}, which involves the consideration, in the solution of the system, of the functions f_0_:[t_0_−δ,t_0_] → R^n^ called initial functions. It can be observed therefore that infinite degrees of freedom exist in the determination of the particular solutions.

In the system described by (3), it is possible to take the initial function f_0_ equal to any y(t), which, in particular, can be a periodic solution of the system for λ_1_ = … = λ_r_ = 0 and t ≤ t_0_.

The initial function will be y^δ^(t):(t_0_−δ,t_0_) → R^n^, with y^δ^(t) = y(t), ∀t ∈ [t_0_ − δ, t_0_].

In the particular case when λ_1_ > 0, λ_2_ = ... = λ_r_ = 0, the first component of the initial function will be *y*_1_^δ^ (*t*) :[*t*_0_ − δ,*t*_0_]→ *R*; to each λ_1_ corresponds a y_1_^δ^ (t_0_ − λ_1_), which is the value of the first component of y in t_0_ − λ_1_; the parameter λ_1_ determines the initial function domain and, given that the solution is periodic, for each different domain of the initial function exists an ordinate value in the origin y_1_^δ^ ( t_0_ − λ_1_) and a corresponding phase-shift. It is observed that y_1_^δ^ ( t_0_ − λ_1_) is the value of the function y_1_^δ^ ( t_0_ − λ_1_) evaluated in t_0_, where h is the initial function with a phase shift of λ_1_.

With this type of systems, it is possible to take into account dynamic behaviors related to parametric variations linked to the independent variable. The parametric variations λ_1_ affect the independent variable which represent time delays and can be related to the phase shifts and the domains of the initial functions. Let us see an example next.

The glycolysis continues to be the best known example of temporal self-organization in metabolic processes, and more than four decades ago the existence of variations in the phase shift values of different metabolites during the glycolytic oscillations was experimentally observed [277,278].

In order to study the repercussion on the dynamic system of phase-shifts, it is suitable to utilize the systems described by differential equations with delay. For example, we can consider a particular ODE-solution to be equal to a periodic solution y(t) of the system (3) for λ_1_ = … = λ_r_ = 0. And we can take this solution as the general initial function f_0_.

As we have seen, each delay time reflects a domain and a phase shift of the initial function. Different domains and phase shifts of the initial functions can be considered in system (3) for each value of the parameter λ linked to the independent variable; and so, particular phase shifted ODE-solutions can be made to correspond to phase-shifted initial functions y^δ^ (t):(t_0_ − δ,t_0_)→R^n^.

In the integration of the delayed functional differential equations system, certain values can be considered for the dependent variables evaluated in t − λ, which correspond to a phase-shifted oscillation of the past. Therefore, it is possible to study if phase-shifted initial functions can be followed (after the corresponding numerical integration) by a mere final phase shift or by a variation in the dynamic behavior of the system.

In this sense, several studies on phase shifts have been carried out in the yeast glycolytic subsystem by means of a delayed differential equations system [279–283] and one of these will be summarized next [281].

In diagram <3>, the main enzymatic processes of yeast glycolysis are represented with the enzymes arranged in series.

In the multienzymatic instability-generating reactive system, it is shown how the metabolite S (glucose), brought into the system at constant speed, is transformed by the first enzyme E_1_ (hexokinase) into the product P_1_ (glucose-6-phosphate). The enzymes E_2_ (phosphofructokinase) and E_3_ (pyruvatekinase) are allosteric, and transform the substrates P′_1_ (fructose 6-phosphate) and P_2_′ (phosphoenolpyruvate) in the products P_2_ (fructose 1-6-bisphosphate) and P_3_ (pyruvate), respectively. The step P_2_ → P_2_′ represents a particular catalytic activity, reflected in the dynamic system by means of a functional variable β′.

It is supposed that a part of P_1_ does not continue in the multienzymatic instability-generating reactive system, q_1_ being the first-order rate constant for the removal of P_1_; likewise q_2_ is the rate constant for the sink of the product P_3_ (which is related with the activity of pyruvate dehydrogenase complex).

In the determination of the enzymatic kinetics of the enzyme E1 (hexokinase) the generic equation of the reaction speed dependent on Glu and MgATP has been used [284]. The speed function of the allosteric enzyme E_2_ (phosphofructokinase) [285,286] was developed in the framework of the concerted transition theory [199]. The reaction speed of the enzyme E_3_, pyruvatekinase, (dependent on ATP, Pyr-P and Fru 1,6-P2) was also constructed on the allosteric model of concerted transition [287].

The main instability-generating mechanism in the glycolytic subsystem is based on the self-catalytic regulation of the enzyme E_2_ (phosphofructokinase), specifically, the positive feed-back exerted by the reaction products, the ADP and fructose-1,6-bisphosphate [60,286,288].

The enzyme E_2_ (Pyruvatekinase) is inhibited by the ATP reaction product [289] and in the first enzyme the influence of the ATP from the final activity of the reactive sequence is considered (the ATP is consumed by E_1_ and recycled by E_3_).

For a spatially homogeneous system, the time-evolution of α, β and γ, which denote the normalized concentrations of P_1_, P_2_ and P_3_, respectively, is described by the following three delay differential equations:

(4) $$\{\begin{array}{l} \frac{\text{d}\alpha }{\text{dt}}={\text{z}}_{1}\sigma_{1}\Phi_{1}(\mu )-\sigma_{2}\Phi_{2}(\alpha ,\beta )-{\text{q}}_{1}\alpha \\ \frac{\text{d}\beta }{\text{dt}}={\text{z}}_{2}\sigma_{2}\Phi_{2}(\alpha ,\beta )-\sigma_{3}\Phi_{3}(\beta ,\beta^{\prime },\mu )\\ \frac{\text{d}\gamma }{\text{dt}}={\text{z}}_{3}\sigma_{3}\Phi_{3}(\beta ,\beta^{\prime },\mu )-{\text{q}}_{2}\gamma \end{array}$$

where

$$\begin{array}{c} \Phi_{1}=\mu {SK}_{D3}/(K_{3}K_{2}+\mu Km_{1}K_{D3}+{SK}_{2}+\mu {SK}_{D3})\\ \Phi_{2}=\frac{\alpha (1+\alpha ){\left(1+d_{1}\beta \right)}^{2}}{L_{1}{\left(1+c\alpha \right)}^{2}+{\left(1+\alpha \right)}^{2}{\left(1+d_{1}\beta \right)}^{2}}\\ \Phi_{3}=\frac{d_{2}\beta^{\prime }{\left(1+d_{2}\beta^{\prime }\right)}^{3}}{L_{2}{\left(1+d_{3}\mu \right)}^{4}+{\left(1+d_{2}\beta^{\prime }\right)}^{4}} \end{array}$$

and

$$\begin{array}{l} \beta^{\prime }=\text{f}(\beta (t-\lambda_{1}))\\ \mu =h(\gamma (t-\lambda_{2})) \end{array}$$

To simplify the model, we did not consider the intermediate part of glycolysis formed by the reversible enzymatic processes. In this way, the functions f and h are supposed to be the identity function:

$$\begin{array}{l} f(\beta (t-\lambda_{1}))=\beta (t-\lambda_{1})\\ h(\gamma (t-\lambda_{2}))=\gamma (t-\lambda_{2}) \end{array}$$

The initial functions present a simple harmonic oscillation in the following form:

$$\begin{array}{l} \alpha_{0}(\text{t})=A+B\text{sin}(2\pi /P)\\ \beta_{0}(t)=C+D\text{sin}(2\pi /P)\\ \gamma_{0}(t)=E+F\text{sin}(2\pi /P)\\ P=534 \end{array}$$

σ_1_, σ_2_ and σ_3_ correspond to the maximum activity of the enzymes E_1_, E_2_ and E_3_ divided by the constants K_m1_, K_m2_ and K_m3_, respectively, (the Michaelis constants of the enzymes E_1_, E_2_ and E_3_); Z_1_ = K_m1_/K_m2,_ Z_2_ = K_m2_/K_m3_ and Z_3_ = K_m3_/K_D3_; L_1_ and L_2_ are the allosteric constant of E_2_ and E_3_; d_1_ = K_m3_/K_D2_, d_2_ = K_m3_/K_D3_ and d_3_ = K_D3_/K_D4_ (K_D3_ and K_D4_ are the dissociation constant of P_2_ by E_3_ and the dissociation constant of MgATP, respectively,); β′ and μ, reflect the normalized concentrations of P_2_′ (Pyr-P) and ATP, respectively; c is the non-exclusive binding coefficient of the substrate; α, β and γ are normalized dividing them by K_m2_, K_m3_ and K_D3_. The values of the different parameters are shown in [281].

Experimental observations, by monitoring the fluorescence of NADH in glycolyzing baker’s yeast under periodic glucose input flux, have shown that the existence of quasiperiodic time patterns is common at low amplitudes of the input flux and chaos emerges at high amplitudes of the input flux [290–292].

In order to simulate these experiments closely, the system can be considered under periodic input flux with a sinusoidal source of substrate S = S′+ *A* sin (ωt) where S′ is the mean input flux rate. The amplitude A and the frequency ω may vary between different simulations.

Assuming the experimental input substrate value of 6 mM/h [287], the normalized mean input flux S′ = 0.033 s^−1^ is obtained after dividing by K_m2_ = 5 × 10^−5^ M, the Michaelis constant of phosphofructokinase for fructose 6-phosphate [293].

Once the values for the parameters are specified and the initial functions are given (see [281,283] for more details), the equations can by solved numerically on a computer.

The numerical results show that in the instability-generating multienzymatic system under a sinusoidal source of substrate quasiperiodic patterns are the most common dynamical behaviors (at low amplitudes of the input flux) and quasiperiodicity routes to chaos can emerge in the biochemical oscillator when the input amplitude is increased. These results are similar to experimental observations [281].

**Figure 5:**
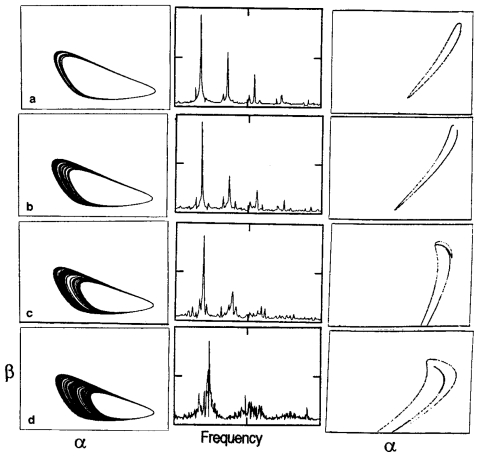
Numerical oscillatory responses of glycolysis under periodic substrate input flux showing a transition sequence to chaos through quasiperiodicity. In the first column are represented the corresponding attractors (projections in two dimensions for the α concentration, x-axis, and the β concentrations, y-axis), power spectra in the second column and Poincaré sections in the α, β plane (third column). Reproduced with permission from Elsevier [283].

Figure 5 displays an example of these transitions to chaos for the following conditions: λ_1_ = 2, λ_2_ = 0.5, q_2_ = 0.069 and S′ = 0.033. So, for A < 0.021, quasiperiodic behaviors emerge in the phase space and two frequencies are present in the oscillations (in Figure 5a, A = 0.01; in Figure 5b, A = 0.017). If both fundamental frequencies have some rational relationship, the system does not explore the whole surface area of the torus but just describes a one-dimensional line corresponding to a periodic or “mode-locked” response (A = 0.016, and A = 0.018). For A = 0.021 (Figure 5c), complex substructures, which show the torus break up being replaced by strange attractors (A/S′ = 0.76) can be observed in the Poincare section and in the power spectra. This route to chaos is also called the Ruelle-Takens-Newhouse route [294,296].

The quasiperiodic route to chaos under periodic substrate input flux is within the range of experimental values [281] and these numerical integrations allow observing some essential aspects of the chaotic behavior emergence in a dissipative biochemical system.

**Figure 6:**
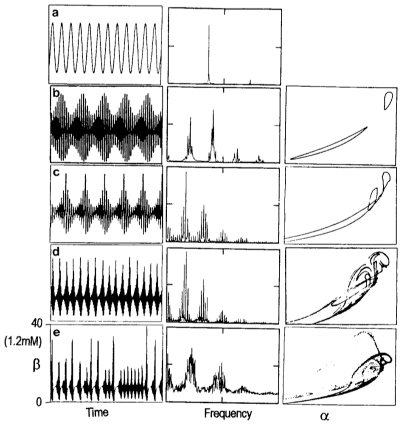
Quasiperiodicity route to chaos under constant substrate input flux. Evolution of (**a**) periodic oscillation, (**b**–**c**) quasiperiodic motion, (**d**) complex quasiperiodic oscillations and (**e**) chaotic responses. (The β concentrations are represented as a function of time). Also shown are the corresponding power spectra (second column) and Poincaré sections (α, β plane). Reproduced with permission from ScienceDirect [281].

Results of the calculations also show a quasiperiodicity route to chaos for a constant input flux (Figure 6). In these new conditions (S = 0.002, λ_1_ = 7, and λ_2_ = 130), the biochemical system exhibits a stable steady state when the control parameter is q_2_ = 0.11. For q_2_ = 0.103, a first Hopf bifurcation introduces a fundamental frequency ω_1_ and a limit cycle appears in the phase space (Figure 6a). For q_2_ = 0.099, a second Hopf bifurcation generates a new fundamental frequency w_2_ causing quasiperiodic behavior (Figure 6b). Above q_2_ = 0.095, complex substructures appears in the torus (Figure 6c,d), and the dynamical behavior originated after the third Hopf bifurcation is not particularly stable and can be perturbed quite easily producing a strange attractor for q_2_ = 0.093 (Figure 6d) [281,283].

Under constant and periodic input flux conditions time delay acts as a source of instability (next to the feedback loops) leading to complex oscillations and transient dynamics in the biochemical system.

Likewise, the numerical study of the glycolytic model formed by a system of three delay-differential equations (4) reveals a notable richness of temporal structures as the three main routes to chaos (the Feigenbaum [283], Intermitency [280] and Quasiperiodicity routes [281,283]) and a multiplicity of stable coexisting states (birhythmicity, trirhythmicity and hard excitation [282,283]).

Stable coexisting states means that under the same parametric conditions the system can exhibit two o more dynamical patterns and any initial metabolite concentrations will eventually lead the system into one of these self-organized behaviors. This dynamical behavior is an important characteristic of the metabolic systems, which has been studied extensively through experiments and numerical simulations [297–301].

Biological examples of metabolic systems with stable coexisting states include genetic switch [302–308], the lactose operon repressor system [309–311], the cell-cycle control [312] and the cellular signal transduction pathways [313–316].

All these dynamical processes (chaos, multiplicity of coexisting states, periodic patterns, bursting oscillations, steady state transitions, *etc.*) show the richness and variety of self-organized phenomena under far-from-thermodynamic equilibrium.

A growing number of works on delayed differential equation systems in biochemical processes are being carried out [317] e.g., it has been studied how delayed repression can induce transient increase and heterogeneity in gene expression [318], the role of delays in the generation of bursting oscillations in neuronal networks [319], the effect of time delays on the robustness of oscillator models [320], and the importance of time delay in biological functions [321].

## 7. Self-Organizations in Stochastic Processes: Genetic Expression

Many metabolic subsystems involve small numbers of molecules causing biochemical processes to be accompanied by fluctuations around the dynamic states predicted by the deterministic evolution of the system. These fluctuations reflect intrinsic molecular noise which may play a very important role in the switching of metabolic dynamics.

Recently, a considerable number of studies in different biochemical processes such as: expression of single genes, gene networks and multi-step regulated pathways allow illustrating the stochastic nature of many metabolic self-organized activities [322–335].

The importance of molecular noise makes us stress that living cells may be also considered stochastic biochemical reactors.

Let us see an example next on the effect of molecular noise on circadian oscillations.

Circadian rhythms govern a wide variety of metabolic and physiological processes in all kinds of cells from prokaryotes to mammals [126,127], and the molecular mechanism of these kinds of metabolic rhythms relies on the negative self-regulatory feedback on gene expression [336–339].

The presence of small amounts of mRNA or proteins in the molecular mechanism of circadian rhythms originates a molecular noise which may become significant and may compromise the emergence of coherent oscillatory patterns [340].

The first model predicting oscillations due to negative feedback on gene expression was proposed by Goodwin [341], at a time when the part played by such a regulatory mechanism in the origin of circadian rhythms was not yet known.

Here is shown a molecular model proposed by A. Golbeter and colleagues for circadian rhythms in *Drosophila* based on negative self-regulatory feedback which has shown robust oscillations in the presence of molecular noise [342].

**Figure 7:**
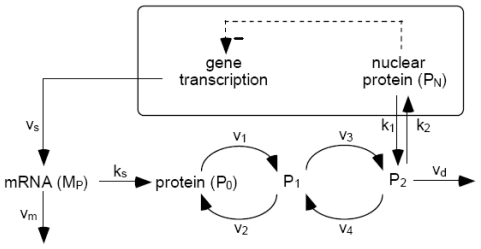
Molecular model for circadian oscillations during genetic expression based on negative self-regulation of the PER gene by its protein product PER. The model incorporates gene transcription into PER mRNA, transport of PER mRNA (M_P_) into the cytosol as well as mRNA degradation, synthesis of the PER protein at a rate proportional to the PER mRNA level, reversible phosphorylation and degradation of PER (P_0_, P_1_ and P_2_), as well as transport of PER into the nucleus (P_N_) where it represses the transcription of the PER gene. Reproduced with permission from PNAS [342].

The core molecular model is schematized in a general form in Figure 7, which is based on the negative feedback exerted by a protein (called clock protein) on the expression of its gene. The nucleocytoplasmic nature of the circadian oscillator implies: gene transcription into Per mRNA (M_P_), transport of per mRNA into the cytosol where it is translated into the clock protein (P_0_) and the mRNA degradation. The synthesis of the (P_0_) PER protein exhibits a rate proportional to the Per mRNA (M_P_) level, and the clock protein can be reversibly phosphorylated from the form P_0_ into the forms P_1_ and P_2_. The phosphorylated form P_2_ can be degraded or transported into the nucleus (P_N_) where it represses the transcription of the gene exerting a negative feedback of cooperative nature.

In the model, the gene presents a maximum rate of transcription v_S_, and the mRNA (M_P_) is degraded by an enzyme with a maximum rate v_m_ and a Michaelis constant K_m_ The kinase and phosphatase involved in the reversible phosphorylation of P_0_ into P_1_, and P_1_ into P_2_, have a maximum rate v_i_ and Michaelis constant K_i_ (*i* = 1,. . .,4). The P_2_ form is degraded by an enzyme with a maximum rate v_d_ and Michaelis constant K_d_, and transported into the nucleus at a rate with an apparent first-order rate constant k_1_. The nuclear form P_N_ is transported into the cytosol with an apparent first-order rate constant k_2_. The negative feedback exerted by P_N_ on gene transcription is described by an equation of the Hill type, in which *n* denotes the degree of cooperativity, and K_I_ is the threshold constant for repression.

The time evolution of the concentrations of mRNA (M_P_) and the various forms of clock protein, cytosolic (P_0_, P_1_ and P_2_) or nuclear (P_N_), is governed by the following system of kinetic differential equations:

$$\{\begin{array}{l} \frac{{\text{dM}}_{\text{p}}}{\text{dt}}={\text{v}}_{\text{s}}\frac{{\text{K}}_{\text{I}}^{\text{n}}}{{\text{K}}_{\text{I}}^{\text{n}}+{\text{p}}_{\text{N}}^{\text{n}}}-{\text{v}}_{\text{m}}\frac{{\text{M}}_{\text{p}}}{{\text{K}}_{\text{m}}+{\text{M}}_{\text{p}}}\\ \frac{{\text{dP}}_{0}}{\text{dt}}={\text{k}}_{\text{s}}{\text{M}}_{\text{p}}-{\text{v}}_{1}\frac{{\text{P}}_{0}}{{\text{K}}_{1}+{\text{P}}_{0}}+{\text{v}}_{2}\frac{{\text{P}}_{1}}{{\text{K}}_{2}+{\text{P}}_{1}}\\ \frac{{\text{dP}}_{1}}{\text{dt}}={\text{v}}_{1}\frac{{\text{P}}_{0}}{{\text{K}}_{1}+{\text{P}}_{0}}-{\text{v}}_{2}\frac{{\text{P}}_{1}}{{\text{K}}_{2}+{\text{P}}_{1}}-{\text{v}}_{3}\frac{{\text{P}}_{1}}{{\text{K}}_{3}+{\text{P}}_{1}}+{\text{v}}_{4}\frac{{\text{P}}_{2}}{{\text{K}}_{4}+{\text{P}}_{2}}\\ \frac{{\text{dP}}_{2}}{\text{dt}}={\text{v}}_{3}\frac{{\text{P}}_{1}}{{\text{K}}_{3}+{\text{P}}_{1}}-{\text{v}}_{4}\frac{{\text{P}}_{2}}{{\text{K}}_{4}+{\text{P}}_{2}}+{\text{v}}_{\text{d}}\frac{{\text{P}}_{2}}{{\text{K}}_{3}+{\text{P}}_{2}}-{\text{k}}_{1}{\text{P}}_{2}+{\text{k}}_{2}{\text{P}}_{\text{N}}\\ \frac{{\text{dP}}_{\text{N}}}{\text{dt}}={\text{k}}_{1}{\text{P}}_{2}-{\text{k}}_{2}{\text{P}}_{\text{N}} \end{array}$$

In cellular conditions, the small amounts of mRNA and proteins provoke an effect of molecular noise on the dynamic behaviors of the system. To perform stochastic simulations of the circadian clock mechanism due to this intrinsic noise, metabolic processes must be decomposed fully into elementary steps (where enzyme-substrate complexes are considered explicitly) and each step is associated with a transition probability proportional both to the numbers of molecules involved and to the biochemical rate constants (the procedure was introduced by Gillespie [343]).

According to this method, the deterministic model schematized in Figure 7 can be decomposed into a detailed reaction system consisting of 30 elementary steps, which occur randomly, with a frequency measured by their probability of occurrence.

The decomposition of the deterministic model into elementary steps, the method of stochastic simulation, and parameter values are listed in *Appendix* of [342].

As an example of the decomposition, steps 1–8, which pertain to the formation and dissociation of the various complexes between the gene promoter and nuclear protein (P_N_), are next shown:

$$\begin{array}{lll} \text{1} & \text{G}+{\text{P}}_{\text{N}}\overset{{\text{a}}_{1}}{\to }{\text{GP}}_{\text{N}} & {\text{w}}_{1}={\text{a}}_{1}\times \text{G}\times {\text{P}}_{\text{N}}/\Omega \\ \text{2} & {\text{GP}}_{\text{N}}\overset{{\text{d}}_{1}}{\to }\text{G}+{\text{P}}_{\text{N}} & {\text{w}}_{2}={\text{d}}_{1}\times {\text{GP}}_{\text{N}}\\ \text{3} & {\text{GP}}_{\text{N}}+{\text{P}}_{\text{N}}\overset{{\text{a}}_{2}}{\to }{\text{GP}}_{\text{N2}} & {\text{w}}_{3}={\text{a}}_{2}\times {\text{GP}}_{\text{N}}\times {\text{P}}_{\text{N}}/\Omega \\ \text{4} & {\text{GP}}_{\text{N2}}\overset{{\text{d}}_{2}}{\to }{\text{GP}}_{\text{N}}+{\text{P}}_{\text{N}} & {\text{w}}_{4}={\text{d}}_{2}\times {\text{GP}}_{\text{N2}}\\ \text{5} & {\text{GP}}_{\text{N2}}+{\text{P}}_{\text{N}}\overset{{\text{a}}_{3}}{\to }{\text{GP}}_{\text{N3}} & {\text{w}}_{5}={\text{a}}_{3}\times {\text{GP}}_{\text{N2}}\times {\text{P}}_{\text{N}}/\Omega \\ \text{6} & {\text{GP}}_{\text{N3}}\overset{{\text{d}}_{3}}{\to }{\text{GP}}_{\text{N2}}+{\text{P}}_{\text{N}} & {\text{w}}_{6}={\text{d}}_{3}\times {\text{GP}}_{\text{N3}}\\ \text{7} & {\text{GP}}_{\text{N3}}+{\text{P}}_{\text{N}}\overset{{\text{a}}_{4}}{\to }{\text{GP}}_{\text{N4}} & {\text{w}}_{7}={\text{a}}_{4}\times {\text{GP}}_{\text{N3}}\times {\text{P}}_{\text{N}}/\Omega \\ \text{8} & {\text{GP}}_{\text{N4}}\overset{{\text{d}}_{4}}{\to }{\text{GP}}_{\text{N3}}+{\text{P}}_{\text{N}} & {\text{w}}_{8}={\text{d}}_{4}\times {\text{GP}}_{\text{N4}} \end{array}$$

The second column lists the sequence of reaction steps, and the probability of each reaction is given in the third column. G denotes the unliganded promoter of the gene, and GP_N_, GP_N2_, GP_N3_ and GP_N4_ are the complexes formed by the gene promoter with 1, 2, 3, or 4 P_N_ molecules, respectively.

The kinetic constants a_j_ and d_j_ = (1,...,4) related to bimolecular reactions are scaled by Ω parameter, which allows modifying the number of molecules present in the system [343,344].

For appropriate parameter values (see appendices in [342]), the numerical integration reveals that the temporal structure of the metabolic system presents sustained circadian oscillations.

**Figure 8:**
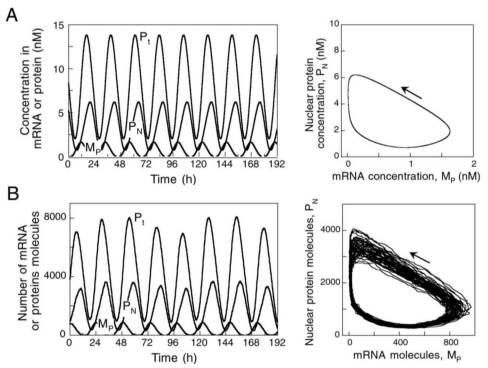
Effect of molecular noise on circadian oscillations during genetic expression. (**a**) Periodic behaviors obtained by numerical integration of the deterministic model in absence of noise. (*Left*) The oscillatory patterns correspond to mRNA (M_P_), nuclear protein (P_N_) and total clock protein (P_t_). (*Right*) Limit cycle obtained as a projection onto the P_N_ – M_P_ phase plane. (**b**) Robust circadian oscillations with a period of 24.4 h produced by the metabolic model in presence of noise. (*Left*) The number of mRNA molecules oscillates between a few and 1,000, whereas nuclear and total clock proteins oscillate in the ranges of 200–4,000 and 800–8,000, respectively. (*Right*) Stochastic simulations of the model yield oscillations that correspond, in the phase plane (P_N_ – M_P_) to the evolution of a noisy limit cycle. Reproduced with permission from PNAS [342].

In Figure 8A, are shown deterministic metabolic rhythms (without noise) of mRNA (M_P_), nuclear (P_N_) and total (P_t_) clock protein under conditions of continuous darkness. The circadian oscillations correspond to the evolution toward a limit cycle, which is shown as a projection of the dynamic behaviors onto the (M_P_,P_N_) phase plane (Figure 8A, right).

Corresponding results from stochastic simulations generated by the model in the presence of noise, for Ω = 500 and *n* = 4 are shown in Figure 8B. The number of mRNA molecules varies in the range of 0–1,000, whereas the numbers of nuclear and total clock protein molecules oscillate in the range of 200–4,000 and 800–8,000, respectively. It can be observed how the molecular noise induces variability in the maxima of the oscillations and, consequently, the trajectory in the phase space shows a thick cloud of points covering the deterministic limit cycle (Figure 8B, right).

The analysis of the molecular model schematized in Figure 7 (see other numerical studies of this system in [342]) allows showing how robust circadian oscillations based on negative self-regulation of gene expression and strengthened by cooperativity can occur even at reduced numbers of mRNA and clock protein molecules of the order of tens and hundreds, respectively.

The analysis of the molecular model schematized in Figure 7 (see other numerical studies of this system in [342]) allows showing how robust circadian oscillations based on negative self-regulation of gene expression and strengthened by cooperativity can occur even at reduced numbers of mRNA and clock protein molecules of the order of tens and hundreds, respectively.

Besides assessing the robustness of circadian oscillations with respect to molecular noise, the analysis of the stochastic model also shows that the persistence of dynamic circadian behaviors is enhanced by the cooperative nature of the gene repression. The role of cooperativity in the circadian metabolic subsystem is supported by the formation of complexes between various clock proteins and this has been observed in several kinds of cells such as *Drosophila*, *Neurospora* and mammals [345–348].

The model represents a prototype for the emergence of self-organized circadian patterns based on negative autoregulatory feedback of gene expression and the numerical results validate the use of deterministic models to study the metabolic mechanism of circadian rhythms and explains why such model provide a reliable picture of the working of circadian clocks in a variety of cells.

Other similar results on circadian clocks with more complex metabolic mechanisms involving a larger number of interacting enzymes can be seen in [349–354].

## 8. Metabolic Attractors

In mathematical studies of metabolic dissipative patterns, self-organization is related to the appearance of attractors in the phase space, which corresponds to ordered motions of the involved biochemical elements.

Phase space is a mathematical object in which all possible states of a system are represented (in form of attractors) and the coordinates correspond to the variables that are required to describe the system.

Attractors in dynamical systems theory provide a way of describing the typical asymptotic orbits. These dynamical trajectories end up and remain in one of the possible attractor states which represent the set of all the possible asymptotic behaviors of the system.

Formally, if for example y(t) is an output activity of a metabolic subsystem, a set A is called an attractor for this subsystem in the following three conditions:

1. It is impossible to go out; in other words, if y(t_0_) is in A for some time t_0_, later y(t) remains in A.
2. There exists a neighbourhood of itself B (basin of attraction) such that for any initial condition in B, the system approaches A indefinitely.
3. A is a compact set; this means it is a closed and bounded set.

Consequently, for a metabolic subsystem under fixed determinate conditions, an attractor is a mathematical dynamical structure that represents the set of all possible asymptotic catalytic behaviors.

There is a great variety of qualitatively different attractors in metabolic subsystems showing the richness of self-organized phenomena under dissipative conditions. Many quantitative studies of metabolic processes are characterized by time series (numerical or experimental) and in certain conditions to investigate some dynamic properties of a biochemical system it is necessary to reconstruct the attractor from these time series.

A method to reconstruct attractors is the time-delay embedding [355]; this technique allows us to establish a phase space representation for time series as a function of the current and of the previous values; for that it requires a delay and an embedding dimension.

Given a time series x(t), t = 1,2,...,N, the m-dimensional return map is obtained by plotting the vector X(t) = [x(t),x(t−τ),x(t−2τ),· · ·,x(t− (m−τ))] where τ is an integer delay.

This converts the dimensional vector x(t), into the m-dimensional vector X(t). The dimension m is known as the embedding dimension, and if m is great enough, the trajectory of X(t) converges to an attractor in the m-dimensional Euclidian space, which is, up to a continuous change of variable, the attractor of the subunit dynamical system.

The election of embedding dimension (m) and delay (τ) is mostly a question of trial and error because although there are criteria, they are not clean-cut [356].

For m election, the method of false nearest neighbors is appropriate. False neighbors are far points in the original phase space with near projections in lower dimensions (see Figure 5 of [357]). The idea is to enlarge m until the number of false neighbors falls to almost zero.

For example, Samll says in [356]: “We can then choose as the embedding dimension m, the minimum value of n for which the proportion of points which satisfy the above condition is below some small threshold.”

What small threshold? Kodba suggests enlarging m until “the fraction of false nearest neighbors convincingly drops to zero [358]”.

How small is “convincingly”? For τ, there is an easy test based on the autocorrelation [359] where the optimal τ would be determined by the time the autocorrelation function first decreases below zero or decays to 1/e.

Alternatively, we can take τ as the first minimum of the mutual information function [356,358]. This criterion is “better” but harder.

A third and easier method is the approximate period: A quarter of the length of the pseudo-period [356].

Time-delay embedding method can be directly applied to a time series by means of software developed with MATLAB.

## 9. Stability in Dynamical Behaviors: The Maximal Lyapunov Exponent

The concept of Lyapunov exponents has been mainly used as a nonparametric diagnosis for stability analysis and for to determine chaotic behaviors, where at least one Lyapunov exponent is positive [360–365].

A positive Lyapunov exponent indicates sensitivity to initial conditions, a hallmark of chaos [366]. By contrast, the leading Lyapunov exponent would be zero for quasiperiodic evolution or when the system is in some sort of steady state mode. A negative Lyapunov exponent is characteristic of a stable fixed point or a stable periodic orbit in the phase space and the dynamical system is insensitive to initial conditions.

The maximal Lyapunov exponent is very useful in testing the existence of chaos and the Wolf algorithm can be used for it [367]. The idea is simple: in mathematics this exponent is a quantity that characterizes the rate of separation of infinitesimally close trajectories belonging to a dynamical system. For example, let us consider a reconstructed attractor (for example, by means of the time-delay embedding method) and define an arbitrary starting point x_0_ lying on it. One should find another point x_0_′ which is close in space but is distant in time to x_0_ :||x_0_ − x_0_′|| = ɛ_0_ ≤ ɛ_min_ and |T(x_0_) − T(x_0_′)| ≥ T_min_. Then trace system dynamics using initial points x_0_ and x_0_′. Then a distance ɛ _0_′ between two trajectories will exceed some value ɛ_max_. Stop and fix the time of tracing T_0_ and the ratio ɛ_0_′/ɛ_0_. After that one should find another starting point x_1_″ which is close to x_1_ and shifted in the direction of the vector x_1_′ – x_1_. Let ||x_1_ – x_1_″|| = *ɛ*_1_. Trace the dynamics of the system using x_1_ and x_1_″ as initial points. Then a distance *ɛ*_1_′ between two trajectories will exceed ɛ_max_. Stop and fix the time of tracing T_1_ and the ratio *ɛ*_1_′/*ɛ*_1_, *etc.*

The Maximal Lyapunov exponent is estimated as

$$\Lambda =\frac{\sum_{\text{k}=0}^{\text{N}}\text{ln}{ɛ}_{\text{k}}^{\prime }/{ɛ}_{\text{k}}}{\sum_{\text{k}=0}^{\text{N}}{\text{T}}_{\text{k}}}$$

where N is the iteration number.

To calculate the maximal Lyapunov exponent, the software developed with MATLAB can be used.

## 10. Long-Term Correlations in Metabolic Activities

In order to study the presence of long-term correlations in metabolic chaotic data, first it is necessary to determinate whether the series is a fractional Gaussian noise (fGn) or a fractional Brownian motion (fBm).

FGn is a stationary stochastic process with the property that the n-th autocorrelation coefficient is given by

(1) $$\rho_{n}=0.5\left\{|n+1{|}^{2H}-2|n{|}^{2H}+|n-1{|}^{2H}\right\}$$

where H is the Hurst coefficient. On the contrary, fBm is a non-stationary, self-similar process, whose first differences form a fGn, that is, taking differences between points sampled at equal intervals a fGn is obtained [368].

Taking into account these concepts and equation (1), fBm is a continuous parameter stochastic process that depends upon a parameter given by the Hurst coefficient H. Thus, it can be denoted the corresponding process by B_H_(t) with 0 ≤ t ≤ ∞; when the independent variable t is sampled at equally spaced times obtaining a discrete fractional Brownian motion. Therefore, fBm is a generalization of Brownian motion in which the increments are normally distributed but they are no longer independent and consequently the process is correlated in time.

FGn and fBm can be distinguished by calculating the slope of the power spectral density plot. The signal is said to exhibit power law scaling if the relationship between its Fourier spectrum and the frequency is approximated asymptotically by S(f) ≈ S(f_0_)/f^β^ for adequate constants S(f_0_) and β. If −1 < β < 1, then the signal corresponds to an fGn. If 1 < β < 3, then the signal corresponds to a fBm [369].

The regression line can be estimated for the pairs (log S(f), log f), where f is the frequency and S(f) the absolute value of the Fourier transform. The β constant is taken to be the opposite of the coefficient of *x* in that regression line.

Most of the physiological time series are fBm, and a number of tools are available for estimating the long-term correlations of an fBm series. The scaled windowed variance analysis is one of the most reliable methods that have been thoroughly tested on fBm signals [370]. In particular, the bridge detrended scaled windowed variance analysis (bdSWV) is usually useful for the analysis of fBm temporal sequences of metabolic activities [371].

This method generates an estimation of the Hurst exponent (H) for each series. In short, for a random process with independent increments, the expected value of H is 0.5. When H differs from 0.5, it indicates the existence of long-term correlations, that is to say, dependence among the values of the process. If H *>*0.5, it is produced by a biased random process which exhibits persistent behavior. In this case, for several previous transitions, an increment on the phase-shift average value implies an increasing trend in the future. Conversely, a previously decreasing trend for a sequence of transitions usually implies a decrease for a similar sequence. Antipersistent behavior is obtained for 0 < H < 0.5, a previously decreasing trend implies a probable increasing trend in the future and an increase is usually followed by decreases [370,371].

According to bdSWV method, if the signal is of the form x_t_, where t = 1,…,*N*, then the following steps are carried out for each one of the window sizes *n* = 2,4,…,*N*/2,*N* (if *N* is not a power of 2, then *n* takes the values 2,4,…,2^k^, where *k* is the integer part of log_2_*N*):

1. Partition of the data points in $\frac{N}{n}$ adjacent non-overlapping windows $\left\{W_{1},\ldots ,W_{\frac{N}{n}}\right\}$ of size *n*, where *W_i_* = {*x*_(_*_i_*_−1)_*_n_*_+1_,..., *x_in_*}. If *N* is not a power of 2 and *N* is not divisible by *n*, then the last remaining points are ignored for this value of *n*. For instance, if *N* = 31 and *n* = 4, the first 28 points are partitioned into seven windows.
2. Subtraction of the line between the first and last points for the points in the n-th window.
3. For each $\text{i}=1,\ldots ,\frac{\text{N}}{\text{n}}$, calculation of the standard deviation SD_i_ of the points in each window, by using the formula:

(5) $${\text{SD}}_{\text{i}}=\sqrt{\sum_{\text{t}=(\text{i}-1)\text{n}+1}^{\text{in}}\frac{{\left({\text{x}}_{\text{t}}-{\bar{\text{x}}}_{\text{i}}\right)}^{2}}{\text{n}-1}}$$

1. where *x̄_i_* is the average in the window W_i_.
2. Evaluation of the average $\bar{SD}$ of the $\frac{N}{n}$ standard deviations corresponding to Equation (5).
3. Observation of the range of the window sizes *n* over which the regression line of $\text{log}(\bar{\text{SD}})$ *versus* log (n) gives a good fit (usually some initial and end pairs are excluded).
4. In this range, the slope of the regression line gives the estimation of the Hurst coefficient *H*.

The empirical range of windows corresponding to step 5) which should be in accordance with the guidelines appearing in [370], and consequently the first two and last three points should be excluded.

The program bdSWV is available on the web of the Fractal Analysis Programs of the National Simulation [372].

Long-term correlations have also been observed in different experimental studies, e.g., the physiological time series [373,374], quantification of DNA patchiness [375], NADPH series [376], DNA sequences [377–379], K^+^ channel activity [380], neural electrical activity [381] and mitochondrial processes [90].

## 11. Measure of Complexity. Kolmogorv-Sinai Entropy

The entropy theory of dynamical systems can be found in many textbooks [382,383]. Roughly speaking, in a biochemical system, the entropy will be highest when all transition states have the same number of possible emergency, and the maximum entropy occurs when any transition pattern could be found with equal probability, therefore the entropy will be lowest and information highest when one pattern or a few patterns are dominant (small number of states with high probabilities).

Entropy is also a useful concept in the study of attractors, which may allow estimating the degree of complexity and information contained in them. More concretely, Kolmogorov–Sinai entropy (K–S entropy) provides a measure of the information and the level of predictability in the attractor and the time series [384]. However, the K–S entropy cannot be computed directly, it can only be approximated. Problems arise when entropy rates have to be estimated from a finite number of observations containing a relatively high noise component.

A practical solution to this problem has been put forward using a developed family of statistics named Approximate Entropy (ApEn) which is a good approximation of the Kolmogorov-Sinai entropy [385].

Formally, given N data points from a time series x(1), x(2),., x(N), two input parameters m and r must be fixed to compute ApEn, denoted precisely by ApEn(m, r, N).

To estimate ApEn, first we form the m dimensional vector sequences X(1)….X(N − m + 1) such that X(i) = (x(i)…..x(I − m + 1)), which represent m consecutive values. Let us define the distance between X(i) and X(j) (d[X(i),X(j)]) as the maximum absolute difference between their respective scalar components and for each X(i) we count the number of j such that d[X(i),X(j)] < r, denoted as N^m^(i) and C_r_^m^(i) = N^m^(i)/(N − m + 1), which measure within a tolerance r the frequency of patterns similar to a given one of window length m.

The average value of C_r_^m^(i) is *φ*^m^(r), which portrays the average frequency of the occurrence that all the m-point patterns in the sequence remain close to each other, and finally

$$ApEn(m,r,N)=\varphi^{m}(r)-\varphi^{\left(m+1\right)}(r)$$

The idea is that ApEn measures the logarithmic likelihood that runs of patterns that are close (within r) for m contiguous observations remain close on subsequent incremental comparisons.

Some Approximate Entropy works can be found in [84,386–390].

ApEn can be calculated with software developed with MATLAB.

## 12. Conclusions

One of the most important goals of contemporary biology is to understand the elemental principles and quantitative laws governing the functional metabolic architecture of the cell.

In this review, I mainly focused on some functional enzymatic structures which allow the temporal self-organization of their metabolic processes.

My aim was to provide an overview of temporal metabolic behaviors, including new examples, some kinds of quantitative mathematical models and non-linear tools for the analysis of dissipative functional enzymatic associations (metabolic subsystems) where oscillatory behaviors may emerge.

From the first studies in 1957 of oscillatory phenomena in fluorescent studies of yeast [190], the number of examples of metabolic oscillatory behavior has grown notably.

Oscillatory phenomena, apart from constituting a singular property that manifests itself at all levels of biological organization, present a great functional significance in enzymatic processes. As recalled in the previous sections, biochemical oscillations constitute in themselves a manifestation of the nonlineal characteristics involved in the metabolic regulation activity.

In the light of the research in course, the biological rhythms that emerge in self-organized biomolecular structures constitute one of the most genuine properties of cellular dynamics; and the rigorous knowledge of their nature and significance may be an essential element in the comprehension of the biological fact at its most basic and elementary levels.

The transition from simple periodic behavior to complex oscillatory phenomena including chaos is often observed in metabolic behaviors. In this sense, the relationship between chaotic patterns and long-term correlations (information correlated in time [165]) is a striking property.

As mentioned above, different studies have evidenced that global cellular enzymatic activities are able to self-organize spontaneously, forming a metabolic core of reactive processes that remain active under different growth conditions while the rest of the metabolic subsystems exhibit structural plasticity. This global and stable cellular metabolic structure (in which also emerge chaotic behaviors) appears to be an intrinsic characteristic common to all cellular organisms [163–165].

The existence of chaotic patterns and long-term correlation properties in the activity of the metabolic subsystems integrated in a stable global functional structure may constitute a biological advantage.

Chaotic patterns exhibit sensitive dependence on initial conditions. Sensitivity means that a small change in the initial state will lead to large changes in posterior system states and the fluctuations of the chaotic patterns are conditioned by the degree of perturbation of the initial conditions. These changes in the system states present exponential divergence, provoking fast separations in the chaotic orbits.

For “slow dynamical systems” the typical time scale of the chaotic fluctuations is on the order of 1 μs [391,392] and in “very fast chaotic systems” the characteristic time scale is on the order of 1 ns [391,393].

Furthermore, different studies have shown that chaos permits fast transmission of information and high efficiency [394].

The existence of chaos (which exhibits long-term correlations) in some functional structures may constitute a biological advantage by allowing fast and specific responses during the adaptation of the metabolic system to environmental perturbations.

For example, calcium plays an important role in the regulation of cell metabolism, modulating many physiological processes [395]. In response to external cellular signals, the cytosolic calcium may exhibit chaotic transitions, which are conditioned by the intensity and type of the perturbation factor [396]. Since many enzymes are modulated by calcium, when intracellular calcium concentration presents chaotic patterns, they exhibit sensitivity to initial conditions and long-term memory properties, which may influence the dynamical activities of the metabolic subsystems, permitting fast and specific metabolic responses during the adaptation to external perturbations.

In this sense, numerous works have shown chaotic behaviors at cellular conditions e.g., in intracellular free amino acid pools [64], respiratory metabolism [79], photosynthetic reactions [94], glycolysis [397], Krebs cycle [398], peroxidase-oxidase reactions [93], membrane potential [399], nuclear translocation of the transcription factor [91], NAD(P)H concentration [400], cyclic AMP concentration [401], ATP concentration [78], intracellular calcium concentration [402].

Since a notable part of the biological temporary processes seem to be chaotic in cell conditions, it can be important to take into account these persistent phenomena in Systems Biology.

A vast amount of new information on structural enzymatic organization and genome dynamics is currently being accumulated, and a great part of the network molecular interactions are perfectly established. Therefore, the real functional structure of the enzymatic associations and genome regulation is an open question to be elucidated through mathematical modeling and numerical analysis.

Within the new area of Systems Biology, quantitative mathematical models, non-linear tools and computational approaches are particularly valuable for exploring dynamic phenomena associated with dissipative metabolic structures, and due to that, these methods will be crucial in making sense of the functional metabolic architecture of the cell.

The comparison of experimental results with numerical analysis calls for more quantitative data on the self-organization of metabolic processes, and these methods will be able to provide an integrative knowledge of the organization of cooperating enzymes into macromolecular complexes and microcompartments with the emergence of temporal metabolic patterns.

This new field fusions several concept and tools from many areas, including: computational intelligence, dynamical systems theory, stochastic processes, nonlinear dynamics and networks theory, among others.

Day by day, Systems Biology is developed as a new methodology about metabolic dynamic processes, which allows explaining how higher-level properties of complex enzymatic processes arise from the interactions among their elemental molecular parts, forming complex spatial structures where singular temporal reactive behaviors emerge.

System Biology will be crucial to the understanding of the functional architecture of the cell.

## Figures and Tables

**Diagram <1>:**
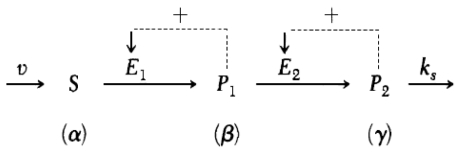


**Diagram <2a>:**
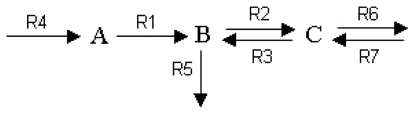


**Diagram <2b>:**
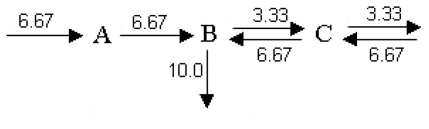


**Diagram <3>:**